# Hyptolactones and Their Biological Potential: A Systematic Review

**DOI:** 10.1021/acs.jnatprod.5c00965

**Published:** 2025-11-25

**Authors:** Felipe Gabriel Henrique Julião, James Almada da Silva

**Affiliations:** † Programa de Pós-graduação em Química, 74391 Universidade Federal de Sergipe, São Cristóvão, Sergipe, 49107-230, Brazil; ‡ Departamento de Farmácia, 74391 Universidade Federal de Sergipe, Lagarto, Sergipe, 49404-044, Brazil

## Abstract

Six-heptyl-5,6-dihydro-2*H*-pyran-2-ones and their analogues, named in this work as hyptolactones, are substances that have great antitumor potential because they are structurally related to pironetin, an important natural product with well-established antitumor effects. To investigate the cytotoxic potential and other biological effects of natural hyptolactones, a systematic review was performed according to the PRISMA guidelines. The search was conducted on databases: PubMed Central, ScienceDirect, Scopus and Web of Science. The authors independently evaluated 272 records after insertion into the Rayyan platform, including 61 articles (Cohen’s Kappa: 0.94), which indicated the existence of 72 hyptolactones originating from plants and 14 from fungi. Of these, 58 demonstrated some type of biological activity, the main ones being cytotoxic, antibacterial and antifungal effects. The SYRCLE tool was used to assess the risk of bias in animal studies. As only the α,β-unsaturated δ-lactones were cytotoxic, it is assumed that the biological effects of the hyptolactones are due to the α,β-unsaturated lactone group, also present in pironetin. Despite promising results, there is a lack of data regarding the biological effects of these lactones, especially *in vivo* models. Thus, scientific evidence of their therapeutic potential motivates more in-depth biological studies, enabling greater expansion of their potential.

## Graphical abstract

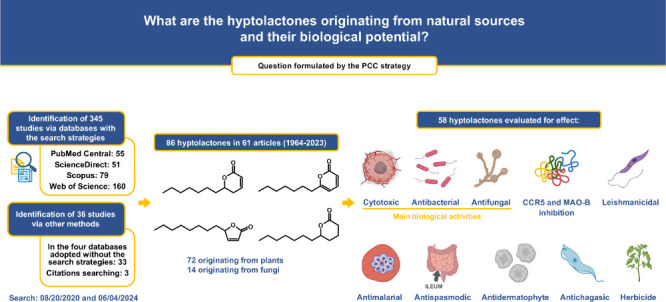

## 1 Introduction

Substances containing the skeleton 6-heptyl-5,6-dihydro-2*H*-pyran-2-one, also called 6-heptenyl-5,6-dihydro-2*H*-pyran-2-one or 6-heptyl-5,6-dihydro-α-pyrone, are secondary metabolites that have the α,β-unsaturated δ-lactone group with a seven-carbon side chain (Figure 1),^1–3^ found in plants of the families Acanthaceae,^4^ Annonaceae,^5^ Aristolochiaceae,^6^ Lamiaceae,^7^ Lauraceae^8^ and Verbenaceae,^9^ and in fungi of the Clavicipitaceae^10^ and Xylariaceae^11^ families.

**Figure 1:**
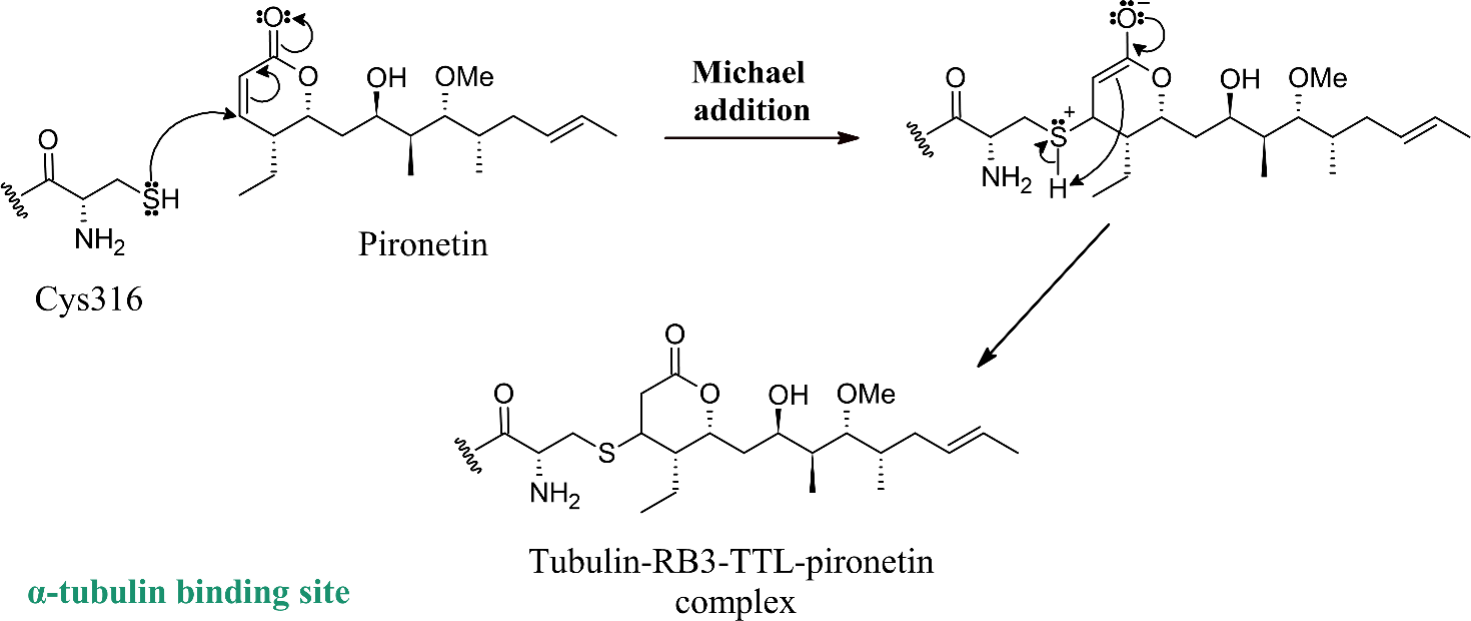
Reaction mechanism of the reaction (Michael addition) between pironetin and the amino acid residue cysteine (Cys316) of α-tubulin to form the tubulin-RB3-TTL-pironetin complex.

Until the development of this work, sixty-six 6-heptyl-5,6-dihydro-2*H*-pyran-2-ones and 20 analogues had been isolated. The analogues included in this study do not have the α,β-unsaturated δ-lactone group, but have extremely similar skeletons: three δ-lactones without ring unsaturations,^1^ 11 α,β,γ,δ-unsaturated δ-lactones^11,12^ and six α,β-unsaturated γ-lactones, also known as 2­(5*H*)-furanones or butenolides.^1,13,14^ Compared to δ-lactones, γ-lactone rings have one less carbon, resulting in an extra carbon in the side chain, as do 6-heptyl-5,6-dihydro-2*H*-pyran-2-ones (Table 1).

**Table 1:**
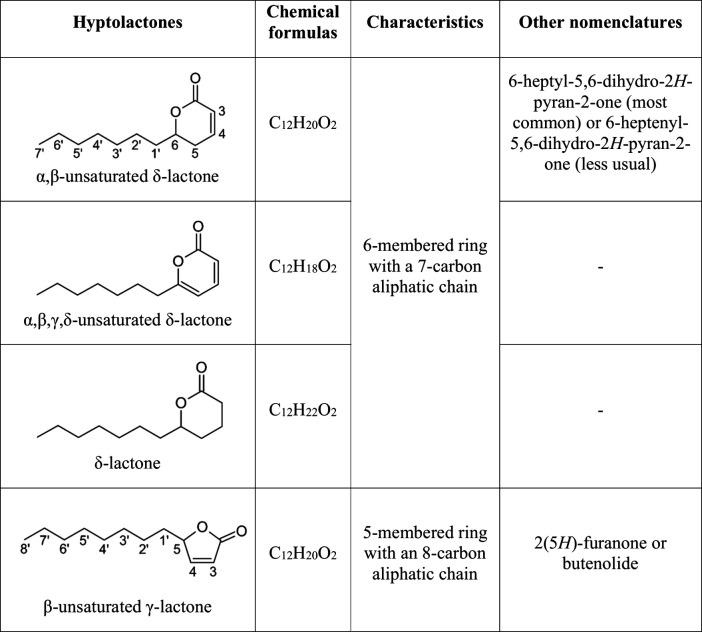
Skeletons of the Chemical Structures, Chemical Formulas, Characteristics, and Other Nomenclatures of Secondary Metabolites Called Hyptolactones

While α,β,γ,δ-unsaturated δ-lactones can be found in fungi of the families Nectriaceae,^12^ Trichocomaceae^15^ and Xylariaceae,^11^ α,β-unsaturated γ-lactones^14^ and δ-lactones without ring unsaturations^1^ can be found in plants of the Lamiaceae family. To group all these secondary metabolites, the nomenclature hyptolactone was assigned to the α,β-unsaturated δ-lactones, δ-lactones, α,β,γ,δ-unsaturated δ-lactones and α,β-unsaturated γ-lactones (Figure 1), a contraction of the words hyptolide, the first 6-heptyl-5,6-dihydro-2*H*-pyran-2-one obtained from a natural source, and lactone. Hyptolide was first isolated from the leaves of *Mesosphaerum pectinatum* (L.) Kuntze (formerly known as *Hyptis pectinata* (L.) Poit.^16^) by Gorter, 1920;^17^ however, only after isolation by Birch and Butler, 1964 ^18^ and Achmad et al., 1987 ^19^ of the leaves of the species was its structure unequivocally elucidated, and its stereochemistry determined.

Hyptolactones are present in many plants that are used for therapeutic purposes in folk medicine, such as *Mesosphaerum pectinatum* and *Hyptis brevipes*. *M. pectinatum* (Lamiaceae) is used to treat infections, inflammations, pain and tumors, with its anti-inflammatory,^20–22^ antimicrobial and cytotoxic against cancer cells^3,7^ scientifically proven. Preliminary biological studies led researchers to investigate its phytochemistry, resulting in the isolation of 17 hyptolactones.^1,3,7,13,14,23^ *H. brevipes* is used to treat infections and tumors, with antibacterial and antifungal activity being scientifically proven.^24^ 15 hyptolactones were isolated out of this species and were related to these biological activities.^25–27^

There are several studies that have proven the biological activities of hyptolactones. Among them, these are antibacterial,^23^ antifungal,^28^ inhibition of the C–C chemokine receptor type 5 (CCR5)^29^ and monoamine oxidase B (MAO-B) enzyme,^30^ leishmanicidal,^5^ antimalarial,^9^ antispasmodic,^31^ antidermatophyte,^32^ antichagasic,^33^ herbicide,^34^ cytotoxic against several human cell lines, such as brain,^15^ cervical,^35^ colorectal,^1^ liver, pancreatic,^36^ renal,^37^ intestine,^15^ larynx,^38^ breast,^3^ nasopharynx,^23^ ovary,^37^ prostate,^39^ lung,^40^ squamous cell carcinoma, fibrosarcoma,^7^ neuroblastoma,^36^ melanoma,^37^ and against murine lymphocytic leukemia cells.^7^ The cytotoxic effect of α,β-unsaturated γ- and δ-lactones was attributed to the electrophilic character of the unsaturated β-carbonyl carbon, with the occurrence of nucleophilic addition reactions (Michael addition) with amino acid residues of proteins in the biological medium.^41–43^ This effect was proven after the assessment of the cytotoxic effect of pironetin (a natural product, isolated from species of the genus *Streptomyces* ^44–46^ ) against cancer cells resistant to drugs such as paclitaxel and vindesine,^47^ and its interaction with α-tubulin. Pironetin is a 6-nonyl-5,6-dihydro-2*H*-pyran-2-one, which forms a complex by covalently binding to α-tubulin by Michael addition, causing apoptosis by disruption of microtubules.^41–43^ It is assumed that the α,β-unsaturated carbonyl bond of the lactone present in pironetin is responsible for its effectiveness against tumor cell lines.^42,43^ It was originally reported that α-tubulin binds to pironetin through the amino acid residue lysine (Lys352);^41^ however, crystallographic studies have demonstrated that the binding can occur through an amino acid residue cysteine (Cys316), due to the proximity of Lys352 to Cys316, which facilitates the transfer of protons, and therefore the occurrence of the reaction. The binding of α-tubulin to pironetin forms a protein complex, called tubulin-RB3-TTL-pironetin, formed by heterodimers of α- and β-tubulin, stem-like protein (RB3), and tubulin tyrosine ligase (TTL).^42,43,48^ Illustrations of the product formed after the Michael addition reaction between pironetin and the amino acid residue Cys316 of α-tubulin are shown in Figure 1, based on the notes of Clayden, Greeves and Warren, 2012,^49^ and in Figure S1 of the Supporting Information.

Despite lacking data, hyptolactones structurally related to pironetin have great therapeutic potential, as previously mentioned regarding the cytotoxic effect on cancer cells. Martínez-Fructuoso et al., 2019 ^1^ demonstrated that hyptolactones that do not have unsaturation in the lactonic ring did not present cytotoxicity in three cancer cell lines, leading us to believe that the therapeutic potential of this class of secondary metabolites is due to the presence of α,β-unsaturated lactone (pharmacophore group), which acts as a strong electrophile in Michael addition reactions, covalently binding to amino acid residues with nucleophilic groups.^42,43^

Unlike cytotoxic activity, there are no scientific reports that correlate the therapeutic potential of hyptolactones to α,β-unsaturated lactones for other proven biological activities. In spite of that, it is likely that, as in cancer cells, this pharmacophore must interact with certain amino acids of vital proteins present in other biological targets. Based on the above, one of the possible ways to ascertain the authenticity of the therapeutic potential of hyptolactones would be through a bibliographic survey of all hyptolactones obtained from natural sources (isolated or synthetic) and their biological potential, using the methodological system of a systematic review, being the first systematic review on this class of secondary metabolites, with this level of coverage.

The study may contribute to the field of natural products by gathering and critically evaluating the available literature on substances with biological potential. The systematization of the chemical diversity and pharmacological activities of hyptolactones may provide a basis for future research in the development of new therapeutic agents, especially for cytotoxic and antimicrobial effects.

## 2 Results and Discussion

### 2.1 Selection of Articles

The present review searched for all hyptolactones and their biological potential. The initial search identified a total of 345 articles: PubMed Central (PMC) (n = 55), ScienceDirect (n = 51), Scopus (n = 79) and Web of Science (n = 160). After removal of duplicates and articles without access, 233 articles were selected for screening stage.

In the first screening, 22 articles were excluded (15 review articles, 3 abstracts, 2 book chapters and 2 notes), with 211 remaining articles to be evaluated in the second screening. Of these, 182 were excluded: 7 articles on syntheses of hyptolactones without demonstration of biological effects and 175 articles on secondary metabolites from other classes. In the third and final screening, the 29 selected articles were evaluated in full text for eligibility, with the purpose of excluding articles that presented divergences in the methodology and results of evaluation of biological activities; however, no article was excluded.

Of the 29 articles included in the study: 24 were on the isolation of hyptolactones, of these, 12 articles did not evaluate biological effects;^4,8,56,57,13,14,50–55^ 11 evaluated biological effects;^5,11,59,12,25–27,31,32,40,58^ and 1 evaluated biological effects, supported by *in silico* studies.^1^ Among the synthesis articles included, 4 evaluated their biological effects.^33,34,38,60^ Finally, 1 article was included that reported its isolations and syntheses, in both obtaining methods, there were demonstrations of biological effects.^7^ Subsequently, the reviewers reached a consensus to include studies using other methods, 36 articles were identified: 3 identified through citations of previously added articles and 33 identified through a search in the four databases adopted (PMC, ScienceDirect, Scopus and Web of Science), without formulated search strategies.

Of the 36 articles identified: 2 were removed because we did not have access, resulting in 34 articles, evaluated in two screenings. Of these, 2 articles were excluded in the first screening because they were syntheses of hyptolactones without demonstrations of biological effects. In the second screening, the 32 selected articles were evaluated in full text for eligibility. Of the 32 articles included in the study, 5 were syntheses of hyptolactones with demonstrations of biological effects^35–37,61,62^ and 27 were isolation articles. Among the isolation articles, 11 did not evaluate biological effects;^6,18,70,19,63–69^ 15 evaluated biological effects;^3,9,72–76,10,15,23,28–30,39,71^ and 1 evaluated biological effects, supported by *in silico* studies.^77^

Finally, of the 272 records independently evaluated by the two reviewers (records from the screening and inclusion stages), 61 articles were added to the systematic review. Of these, 23 are isolated without demonstration of biological effects; 26 are isolation with demonstrations of biological effects; 2 are isolation with demonstrations of biological effects, supported by *in silico* studies; 9 are syntheses with demonstrations of biological effects; and 1 isolation and synthesis, in both obtaining methods, with demonstrations of biological effects. A flowchart illustrating the progressive stages for inclusion of articles in the systematic review, prepared in accordance with the PRISMA 2020 statement,^78,79^ is shown in Figure 2.

**Figure 2:**
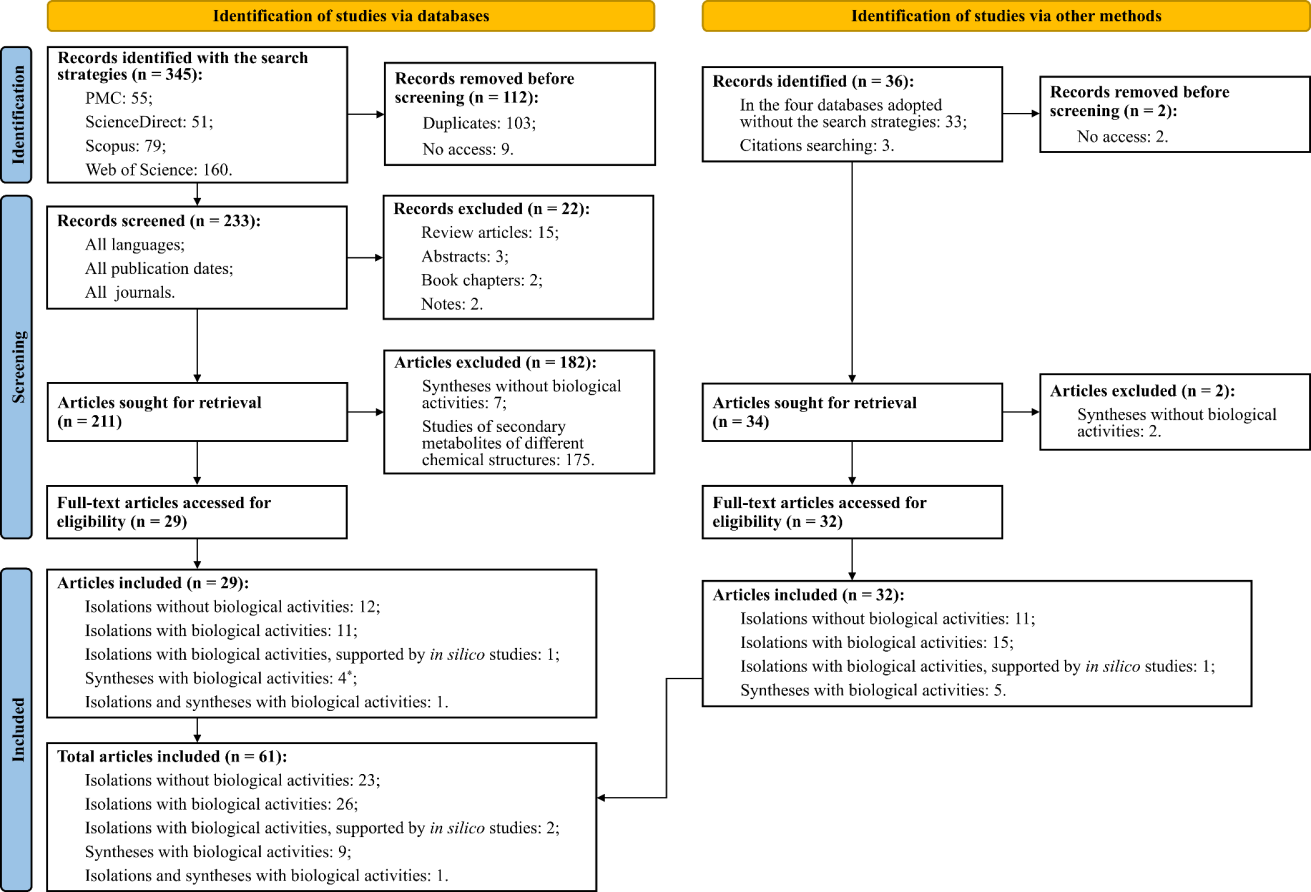
PRISMA 2020 flowchart with the different phases of the systematic review to include studies with hyptolactones. *In one of the articles, the authors did not report how they obtained the hyptolactones evaluated biologically, it is assumed that their attainments occurred by syntheses, due to the type of content covered in the study.^38^

It was noted that the 61 articles included were all written in English and published between the years 1964–2023. It can be observed that in 2020 there was the largest number of publications on hyptolactones. A representation of the annual estimate of the articles included in the study is shown in Figure 3.

**Figure 3:**
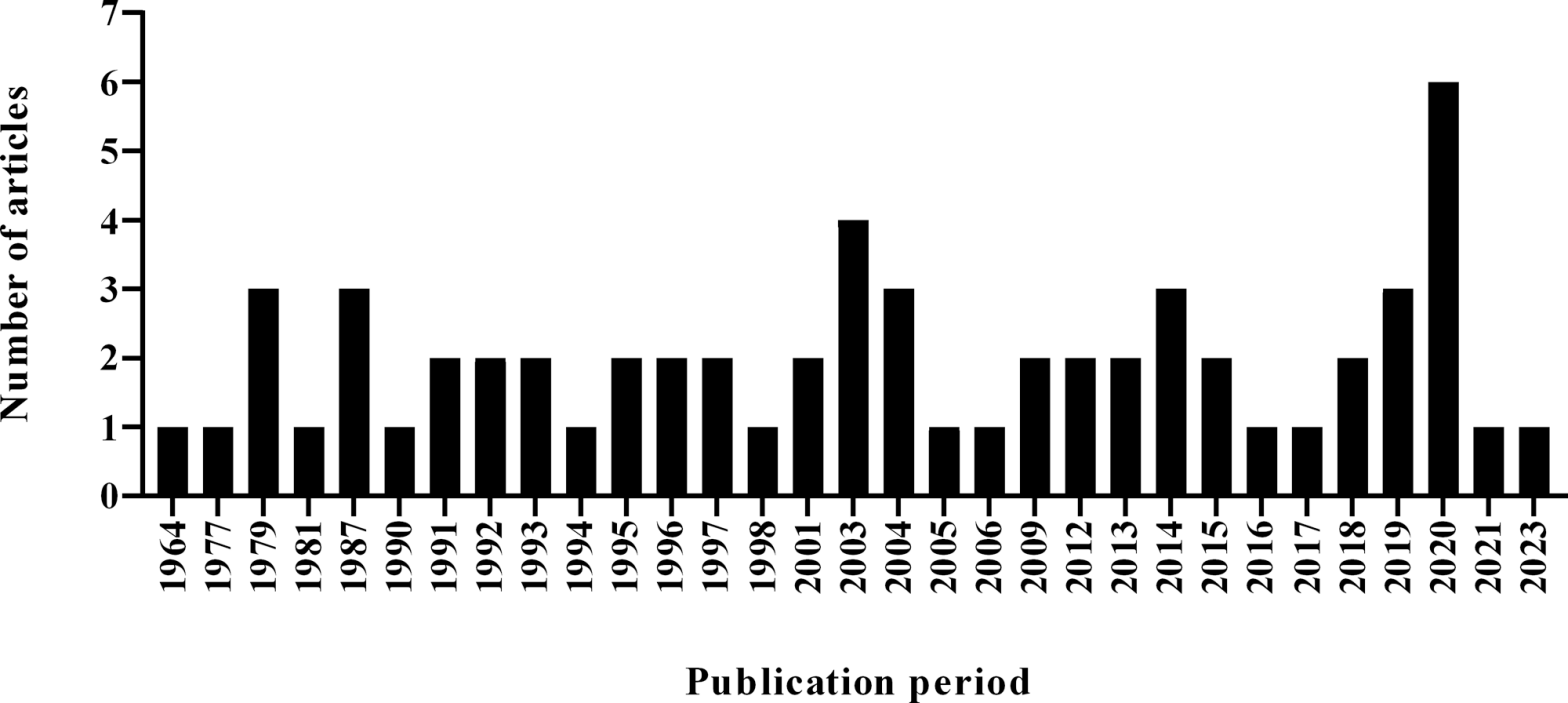
Number of articles published between 1964 and 2023 of the 61 articles involving hyptolactones, added in the systematic review.

### 2.2 Studies Characteristics

After the article inclusion stage, it was found that there are 86 secondary metabolites from the hyptolactone class. Of the 86 secondary metabolites, 72 were found in plants of the families Acanthaceae,^4^ Annonaceae,^5^ Aristolochiaceae,^6^ Lamiaceae,^7^ Lauraceae,^8^ and Verbenaceae,^9^ and 14 were found in fungi of the families Clavicipitaceae,^10^ Nectriaceae,^12^ Trichocomaceae^15^ and Xylariaceae.^11^

Hyptolactones isolated from plants were: anamarine (**1**),^63^ argentilactone (**2**),^72^ boronolide (**3**),^67^ brevipolides A-O (**4–18**),^25–27,29^ deacetylboronolide (**19**),^4,65^ deacetylumuravumbolide (**20**),^4,53,65^ hyptenolide (**23**),^31^ hyptolide (**24**),^76^ lippialactone (**25**),^9^ monticolides A-F (**26–31**),^1,57^ monticofuranolide A (**78**),^14^ neohyptolide (**32**),^59^ olguine (**33**),^64^ pectinolides A-E (**34–38**), pectinolide F (**84**), pectinolides G-H (**79–80**), pectinolides I–K (**39–41**), pectinolides L-M (**85**-**86**), pectinolides N–P (**81–83**), pectinolide J′ (**42**),^1,3,7,13,14,23^ spicigera lactone (**43**),^69^ spicigerolide (**44**),^58^ synargentolides A-E (**45–49**),^56^ syndenolido (**50**),^52^ synparvolidos A-C (**51–53**),^55^ synrotolide (**54**),^50^ umuravumbolide (**55**),^4,65^ 1′,2′-dideacetylboronolide (**57**),^4,66^ 4-deacetoxy-10-*epi*-olguine (**58**),^40^ 10-*epi*-olguine (**59**),^39^ 6*R*-[5*R*,6*S*-(diacetyloxy)-1*S*,2*R*-(dihydroxy)-3*E*-heptenyl]-5,6-dihydro-2*H*-pyran-2-one (**60**), 6*R*-[5*R*,6*S*-(diacetyloxy)-1*R*-(hydroxy)-2*R*-(methoxy)-3*E*-heptenyl]-5,6-dihydro-2*H*-pyran-2-one (**61**), 6*R*-[1*R*,2*R*,5*R*,6*S*-(tetracetyloxy)-3*E*-heptenyl]-5,6-dihydro-2*H*-pyran-2-one (**62**),^40^ (6*R*)-(5′-oxohepten-1′*E*,3′*E*-dienyl)-5,6-dihydro-2*H*-pyran-2-one (**63**), (6*S*)-(5′-oxohepten-1′*Z*,3′*E*-dienyl)-5,6-dihydro-2*H*-pyran-2-one (**64**),^5,80^ 6*R*-[2*R*,4*S*,6*S*-(triacetoxy)-heptyl]-5,6-dihydro-2*H*-pyran-2-one (**65**)^8,81^ and (2′-acetoxy)-6-hept-4-enyl-5,6-dihydro-2*H*-pyran-2-one (**66**).^54^

Hyptolactones isolated from fungi were: fupyrone A (**67**), fupyrone B (**68**),^12^ gamahonolide A (**21**), gamahonolide B (**22**),^10^ xylariaopyrone A (**69**), xylariaopyrone B (**56**), xylariaopyrones C-G (**70–74**),^11,30^ 6-(2′*R*-hydroxy-3′*E*,5′*E*-diene-1′-heptyl)-4-hydroxy-3-methyl-2*H*-pyran-2-one (**75**), 6-(2′*S*-hydroxy-5′*E*-ene-1′-heptyl)-4-hydroxy-3-methyl-2*H*-pyran-2-one (**76**) and 6-(2′*S*-hydroxy-1′-heptyl)-4-hydroxy-3-methyl-2*H*-pyran-2-one (**77**).^15^ Of all these hyptolactones, 66 are α,β-unsaturated δ-lactones and 20 are analogous lactones (Figure 4).

**Figure 4:**
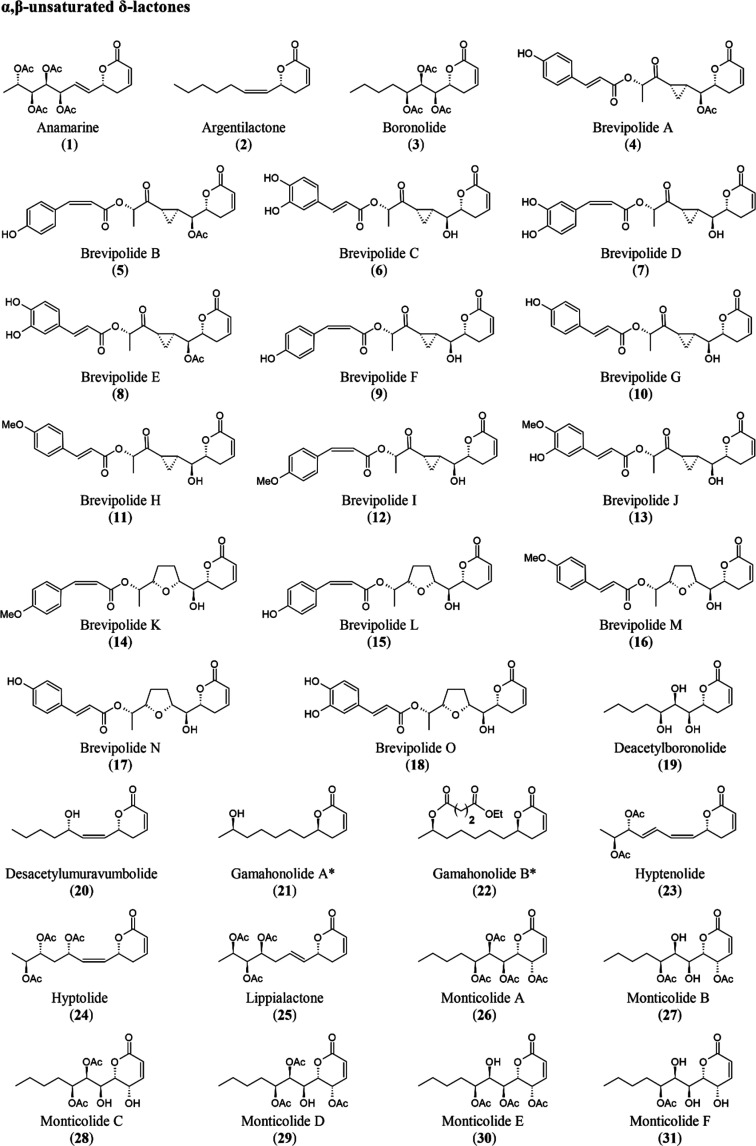

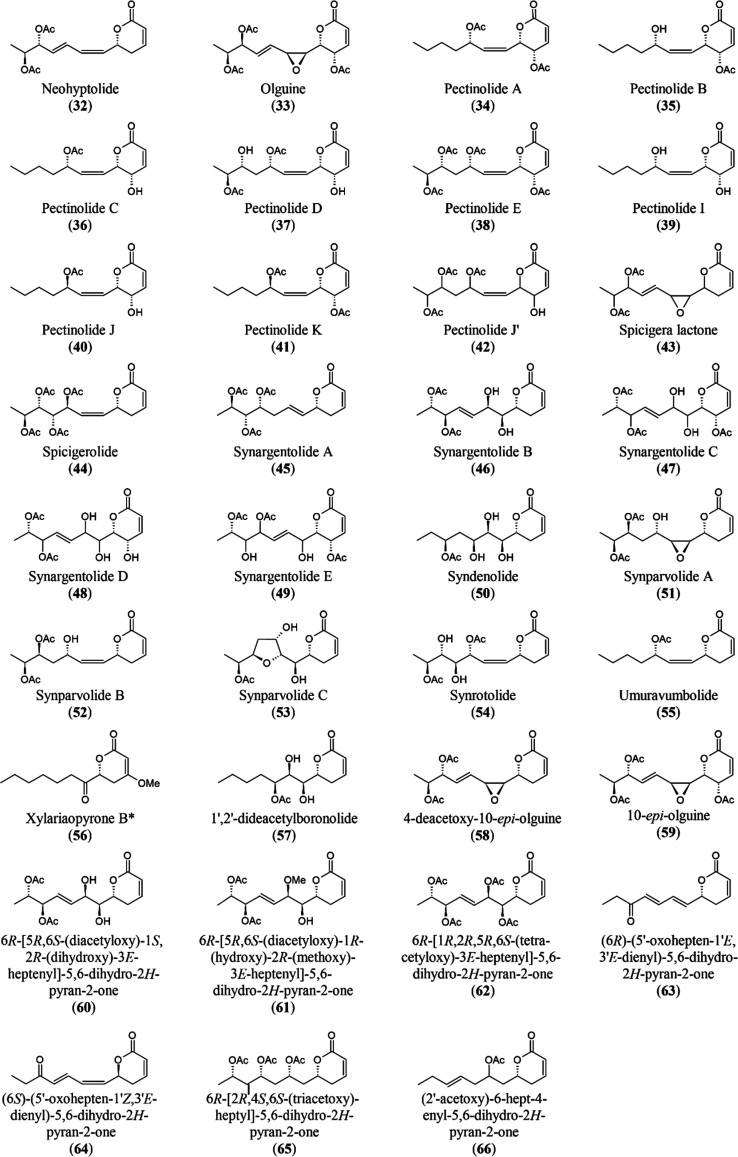

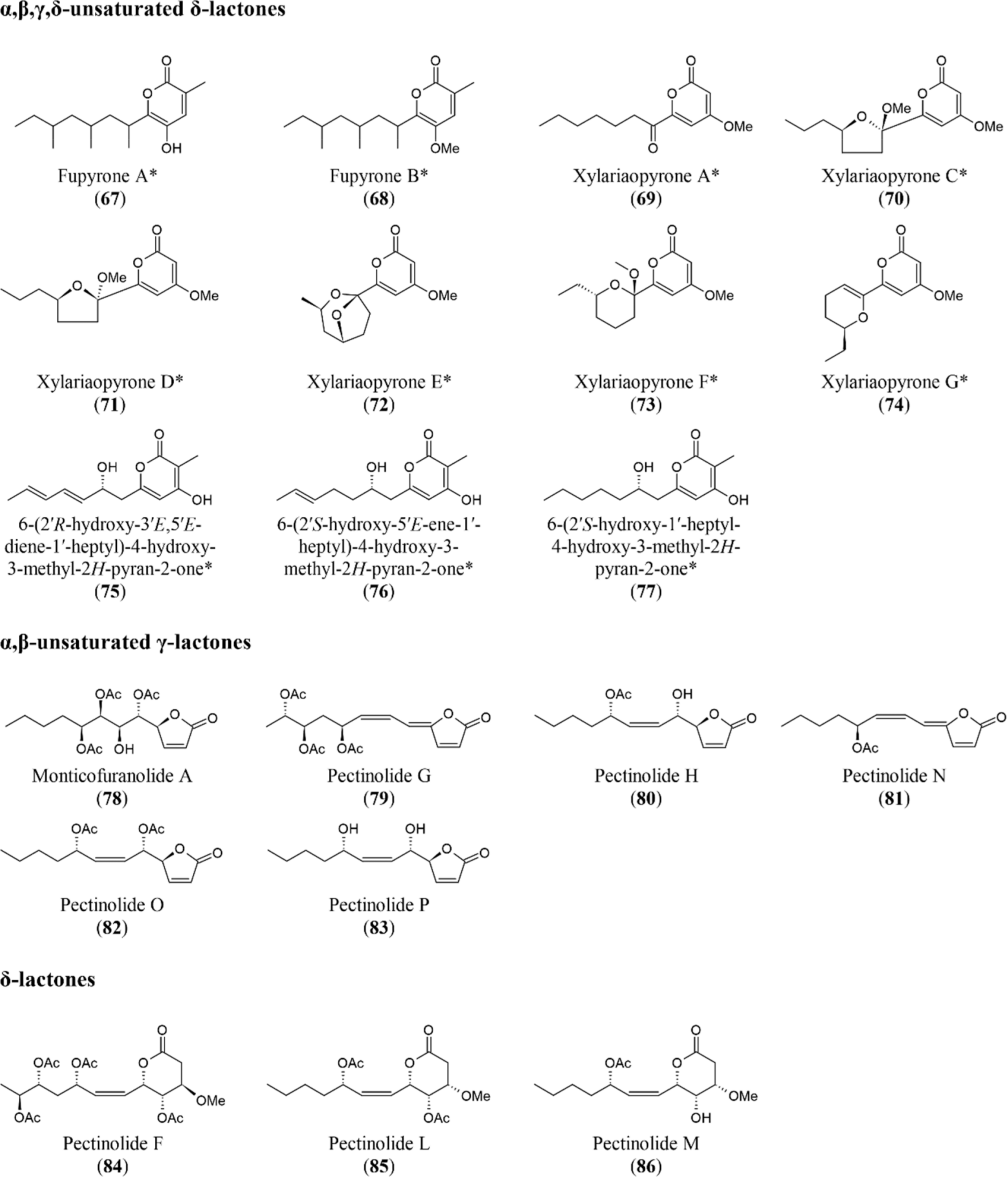
Representation of the chemical structures of the 86 hyptolactones. *Isolated from fungi.

Hyptolactones are present mainly in plants of the Lamiaceae family, corresponding to 67 of the 72 hyptolactones isolated from plants. They are **1–20**, **23**, **24**, **26–55**, **57–62**, **78–86**. Substance **2** was also found in the Aristolochiaceae family, and together with substances **63** and **64**, was found in the Annonaceae family. Substances **19**, **20**, **55** and **57** were also found in the Acanthaceae family. Substances **10**, **11** and **12** were also found in the Verbenaceae family, as well as substance **25**. In the Lauraceae family, substances **65** and **66** were found. Of the hyptolactones present in fungi, substances **21** and **22** were found in the Clavicipitaceae family, **67** and **68** in the family Nectriaceae, **56**, **69–74** in the Xylariaceae family, and **75**, **76** and **77** in the Trichocomaceae family.

### 2.3 Biological Effects of Hyptolactones

Of the 86 hyptolactones, 58 had their biological potential evaluated (45 originating from plants and 13 originating from fungi) (Table 2). Among those originating from plants, there are 42 α,β-unsaturated δ-lactones: **2–18**, **20**, **23–27**, **32**, **34–36**, **38–42**, **44**, **54**, **55**, **58–64**; 2 δ-lactones: **85** and **86**; and 1 α,β-unsaturated γ-lactone: **80**. Among those originating from fungi, there are 2 α,β-unsaturated δ-lactones: **21** and **56**; and 11 α,β,γ,δ-unsaturated δ-lactones: **67–77**. Those originating from fungi included in the study were obtained in smaller quantities, consequently, a smaller variety of biological tests was carried out. A flowchart illustrating the total number of hyptolactones and their biological effects present in the 61 articles included in the systematic review is show in Figure S2 of the Supporting Information.

**Table 2:** Biological Activities and Respective Percentages of the 58 Hyptolactones

Biological activities	Hyptolactones	Studies
Originating from Plants (45 Hyptolactones)		
Cytotoxic: 38/58 (65.5%)	**2**	de Fatima et al., 2004 ^37^
	**4–9**	Deng et al., 2009 ^25^
	**10**	Deng et al., 2009,^25^ Suárez-Ortiz et al., 2013 ^26^
	**11–13**	Suárez-Ortiz et al., 2013 ^26^
	**14–18**	Suárez-Ortiz et al., 2017 ^27^
	**20** and **55**	Sabitha et al., 2012 ^35^
	**26**, **27**, **39–41**, **85** and **86**	Martínez-Fructuoso et al., 2019 ^1^
	**3**, **34–36**	Pereda-Miranda et al., 1993 ^7^
	**24**, **38** and **42**	Santana et al., 2019 ^3^
	**80**	Fragoso-Serrano et al., 2005 ^23^
	**44**	Pereda-Miranda et al., 2001 ^58^
	**54**	Sabitha et al., 2014 ^36^
	**58** and **62**	Pereda-Miranda et al., 1990 ^40^
	**59**	Lu et al., 1997 ^39^
Antibacterial: 10/58 (17.2%)	**24**	Pereda-Miranda et al., 1993 ^7^
	**32**	Rahman and Gibbons, 2015 ^59^
	**34–36** and **80**	Fragoso-Serrano et al., 2005 ^23^
	**58**	Rahman and Gibbons, 2015 ^59^
	**60–62**	Rojas et al., 1992 ^71^
CCR5 inhibition: 3/58 (5.2%)	**10–12**	Hegde et al., 2004 ^29^
Antifungal: 5/58 (8.6%)	**24**, **34–36**	Pereda-Miranda et al., 1993 ^7^
	**2**	Araújo et al., 2016 ^74^
Antichagasic: 1/58 (1.7%)		de Fátima et al., 2006 ^33^
Antidermatophyte: 1/58 (1.7%)		de Oliveira et al., 2004 ^32^
Antimalarial: 2/58 (3.4%)		Carmona et al., 2003 ^5^
	**25**	Ludere et al., 2013 ^9^
Herbicide: 1/58 (1.7%)	**3**	Yamauchi et al., 2012 ^34^
Leishmanicidal: 3/58 (5.2%)	**2**	Waechter et al., 1997 ^72^
	**63** and **64**	Carmona et al., 2003 ^5^
Antispasmodic: 1/58 (1.7%)	**23**	Costa et al., 2014 ^31^
Originating from Fungi (13 Hyptolactones)		
Cytotoxic: 7/58 (12.1%)	**56**, **69–71**	Guo et al., 2018 ^11^
	**75–77**	Zhao et al., 2018 ^15^
Antibacterial: 12/58 (20.7%)	**56**, **69–71**	Guo et al., 2018 ^11^
	**72–74**	Yang et al., 2020 ^30^
	**67** and **68**	Gao et al., 2020 ^12^
	**75–77**	Zhao et al., 2018 ^15^
Antifungal: 4/58 (6.9%)	**21**	Koshino et al., 1992 ^10^
	**75–77**	Zhao et al., 2018 ^15^
MAO-B inhibition: 3/58 (5.2%)	**72–74**	Yang et al., 2020 ^30^

Table S1 of the Supporting Information provides taxonomic information, biological effects, biological models and doses/concentrations/potency of the 58 hyptolactones that showed biological activities. The results presented the biological responses in the form of inhibitory concentration at 50% (IC_50_), effective dose at 50% (ED_50_), growth inhibition at 50% (GI_50_), minimum inhibitory concentration (MIC), minimum bactericidal concentration (MBC) and minimum fungicidal concentration (MFC). Information regarding the family and species of each secondary metabolite helped us observe which hyptolactones were from species with common taxonomies. Taxonomy made it easier to determine how many hyptolactones were isolated from plants and fungi, and in which species and families these substances were mostly found.

#### 2.3.1 Cytotoxic Activity against Cancer Cells

Cytotoxic activity is the predominant activity in the hyptolactonas class (77.6%), mainly among the hyptolactones originating from plants (65.5%). Of the 45 hyptolactones that were subjected to *in vitro* cytotoxic assays, 36 are α,β-unsaturated δ-lactones, 6 are α,β,γ,δ-unsaturated δ-lactones, 2 are δ-lactones and one is α,β-unsaturated γ-lactone. Only the α,β-unsaturated δ-lactones showed cytotoxic effects, as demonstrated in Figure S3 (Supporting Information). For the δ-lactones without unsaturation (**85** and **86**), the absence of activity was expected, since they do not contain the α,β-unsaturated lactone group in their structures (Table S1), which is point to as the pharmacophoric group for hyptolactones.

Among the α,β-unsaturated δ-lactones, only compounds **6–8** showed no cytotoxic effect. In contrast, **2**, **3**, **24**, **34–36**, and **58** were the hyptolactones with cytotoxic effects in the greatest number of cell lines. For classification purposes, a cutoff point of 10 μM was adopted as the criterion for cytotoxicity. Thus, compounds with IC_50_ values ≤ 10 μM were considered cytotoxic/active, while values above 10 μM indicated an absence of cytotoxicity. Except for kidney and ovarian cancer cells, the majority of hyptolactones exhibited cytotoxicity against the evaluated cell lines, with IC_50_ values ranging between 0.2 and 10 μM.

Some hyptolactones showed divergent results when evaluated in the same cell line. For example, compound **10** exhibited IC_50_ values of 3.6 ^25^ and 13.2 ^26^ μM against the MCF-7 breast cancer cell line. The discrepant IC_50_ values reported for the same compound in the same cell line may be attributed to methodological differences between studies. Among the factors influencing the discrepancy in values, the following stand out: the type of viability assay, compound exposure time, initial cell density, culture medium and serum percentage, range of tested concentrations, and the statistical method used for dose–response curve fitting. Thus, without standardization of experimental conditions, numerical IC_50_ values are not directly comparable and should be interpreted with caution.

In contrast, compound **24**, for example, exhibited an IC_50_ of 9.8 μM against the KB nasopharyngeal cancer cell line in different studies,^7,38^ demonstrating consistent methodological rigor. Despite the limitations that introduce bias in certain studies, the search for new cytotoxic agents from natural sources remains relevant, as natural products offer broad structural and functional diversity. This chemical richness has historically contributed to drug discovery, representing a promising strategy for identifying compounds with greater efficacy against cancer cells, as exemplified by paclitaxel, a natural-origin anticancer compound.^82,83^

The α,β,γ,δ-unsaturated δ-lactones, the α,β-unsaturated γ-lactone, and δ-lactones without unsaturation in the ring did not present cytotoxic effect, which leads us to believe that the presence of α,β-unsaturation in the δ-lactone rings is very important for this biological effect, favoring molecular recognition and allowing a ligand-target interaction suitable for the induction of cytotoxicity. The α,β-unsaturated δ-lactones are structurally related to pironetin, a natural product with cytotoxic activity, which has the α,β-unsaturated δ-lactone group in its structure. Bañuelos-Hernández et al., 2014 ^84^ carried out a molecular docking study to identify possible binding modes between pironetin and α-tubulin in the formation of the pironetin-tubulin complex. The result indicated that there are hydrogen bonding interactions with pironetin through the amino acid residues of asparagine (Asn249 and Asn258) and lysine (Lys352). It was also observed that the amino group of Lys352 and the β-carbon of the lactone α,β-unsaturation were close enough (3.69 to 5.38 Å) to favor a Michael addition (E_f_ ≈ −7.00 kcal/mol).

Martínez-Fructuoso et al., 2019 ^1^ carried out a molecular docking study with α,β-unsaturated δ-lactones: monticolides **26** and **27**, pectinolides **36** and **41**, and α-tubulin. The interaction occurred mainly through hydrogen bonds with the amino acid residues of phenylalanine (Phe351), valine (Val353) and lysine (Lys352) in the same α-tubulin unit proposed for the pironetin interaction, with similar binding energies. For the complex with substance **36**, the distance between the amino group of Lys352 and the β-carbon of α,β-unsaturated δ-lactone was 5.10 Å, which favors Michael addition (E_f_ = −5.82 kcal/mol). Other interactions were observed: three hydrogen bonds between the oxygen of the δ-lactone ring and the amidic hydrogen of Val353, carbonyl of the acetoxy residue at C-3′ in the side chain with the amidic hydrogen of Phe351, and the carbonyl of the acetoxy residue at C-5 with the amide hydrogen of Lys352 (Figure S4). The other substances exhibited similar energy values of interactions observed for substance **36**. Molecular docking analysis confirmed that the α,β-unsaturated lactone group of hyptolactones is important for the biological effect and that they can form a covalent bond with Lys352 of α-tubulin via Michael addition.^1^

Despite having α,β-unsaturation in the lactone ring, the absence of cytotoxic activity in α,β,γ,δ-unsaturated δ-lactones and α,β-unsaturated γ-lactones may be related to other structural characteristics, such as ring size, distribution of double bonds, and substituent groups, which may avoid interaction with the biological target. As reported, the α,β-unsaturated group acts as the electrophile reacting with nucleophilic amino acid residues to induce cytotoxicity. It is hypothesized that α,β,γ,δ-unsaturated δ-lactones contain conjugated double bonds within the ring, which reduces the electrophilicity of the α,β-unsaturated center. Meanwhile, α,β-unsaturated γ-lactones (butenolides) possess a smaller lactone ring, altering ring strain and spatial orientation. These modifications reduce the compatibility between the pharmacophore and cellular targets. Saturated δ-lactones lack the pharmacophore, making them chemically incapable of forming covalent bonds with cellular nucleophiles. The hyptolactonas that contain the α,β-unsaturated δ-lactones group therefore have enormous cytotoxic potential when compared to other natural substances that contain lactone groups, in addition to the new cytotoxic mechanism of action presented by pectinolides. The possible Michael addition reactions for hyptolactones are illustrated in Figure 5.

**Figure 5:**
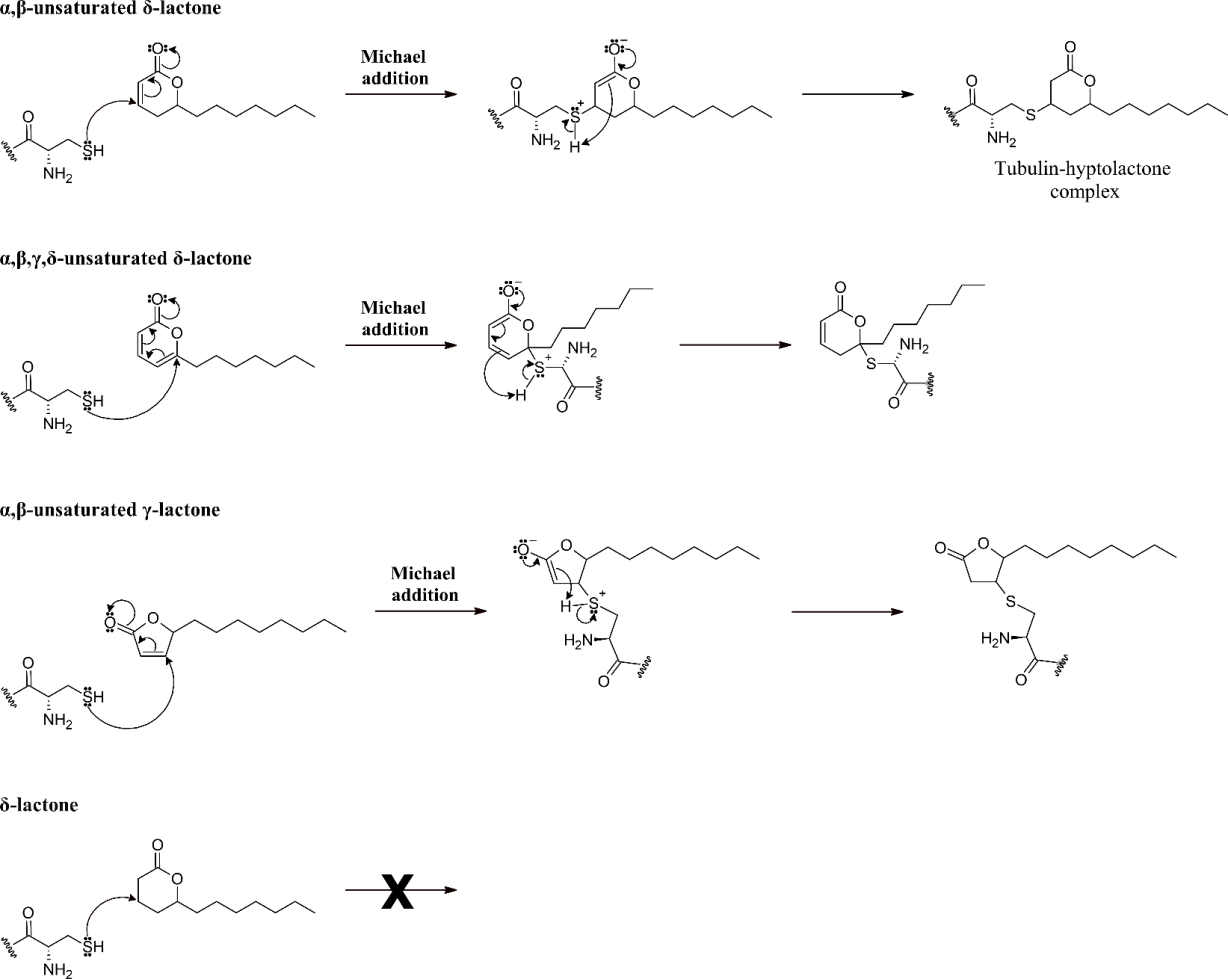
Possible Michael addition reactions between hyptolactones and the cysteine residue of α-tubulin lead to the formation of the hyptolactone-pironetin complex.

#### 2.3.2 Antibacterial and Antifungal Activity

Antibacterial activity was the second most common biological activity, with 22 hyptolactones evaluated (37.9%). Among the 22 hyptolactones that were subjected to *in vitro* antibacterial tests, 10 are α,β-unsaturated δ-lactones (**24**, **32**, **34**, **35**, **36**, **56**, **58**, **60**, **61** and **62**) and 11 are α,β,γ,δ-unsaturated δ-lactones (**67**-**77**, **80**). Among them, there are 11 hyptolactones originating from fungi: one α,β-unsaturated δ-lactone, and 10 α,β,γ,δ-unsaturated δ-lactones (Figure S5). All hyptolactones exhibited biological effects against Gram-positive and Gram-negative bacteria.

According to Ríos and Recio, 2005,^85^ substances present strong antimicrobial activity when the MIC is lower than 10 μg/mL, and inactive when the MIC is greater than 100 μg/mL. Overall, the lactones showed activity between 10 and 100 μg/mL, and therefore we will consider that this compounds presented moderate antimicrobial effect against the evaluated bacteria. The hyptolactone **34** was the only lactone that exhibited strong antibacterial activity against *B. subtilis*.

Despite generally exhibiting moderate activity, the hyptolactones were able to inhibit the growth of bacteria of major clinical importance, as *E. coli*, *K. aerogenes*, *S. Typhi*, and especially *S. aureus*, a pathogen with significant public health impact and disease severity, particularly the Methicillin-Resistant *Staphylococcus aureus* strain (MRSA). Hyptolactones **32**, **58**, and **80** showed the lowest MICs against MRSA. The MIC values for compound **58** were similar across multiple *S. aureus* strains, including the standard strain, clinical strain, laboratory strain, and epidemic (MRSA) strain.^59^ These results indicate that the compound’s antibacterial activity is not affected by resistance mechanisms, underscoring its potential as a broad-spectrum agent against both laboratory strains and clinically relevant strains.

Antifungal activity was the third most common biological activity, with 9 hyptolactones evaluated *in vitro* (15.5%): 6 are α,β-unsaturated δ-lactones (**2**, **21**, **24**, **34**, **35** and **36)** and 3 are α,β,γ,δ-unsaturated δ-lactones (**75**, **76** and **77**). Among them, there are 4 hyptolactones originating from fungi: one is α,β-unsaturated δ-lactone, and 3 are α,β,γ,δ-unsaturated δ-lactones.

Substance **2** exhibited antifungal activity against *Paracoccidioides lutzii* (Pb01), inhibiting growth, the mycelium-to-yeast transition, and isocitrate lyase (PbICL). Yeasts of the species were sensitive to the substance **2** at a concentration of 18 μg/mL, for the mycelium-to-yeast transition, while the MIC was 72 μg/mL. Substance **2** inhibited the activity of recombinant and native PbICL, with IC_50_ of 5.6 μg/mL for the recombinant enzyme and IC_50_ of 9.7 and 15.5 μg/mL for the native enzyme.^77^

Proteomic analysis of *P. lutzii* yeast treated with substance **2** (9.0 μg/mL) revealed effects on enzymes of glycolysis, Krebs, and glyoxylate cycles, indicating disruption of key energy-producing pathways.^73^ Substance **2** was evaluated against *Paracoccidioides* species (*P. lutzii*, *P. brasiliensis*, *P. americana*, and *P. restrepiensis*) with MIC and MFC values of 4.5–36 μg/mL. At 36 μg/mL, it inhibited malate dehydrogenase, citrate synthase, and pyruvate dehydrogenase, induced reactive oxygen species, and impaired cell wall polymer biosynthesis in *P. brasiliensis* (Pb18).^28,74^ The results suggest that the substance has great potential as antifungal agent, not only by inhibiting microbial growth, but also as the only evaluated lactone to have its potential assessed in inhibiting cellular and enzymatic processes of an antimicrobial.

Substances **24**, **34**, **35** and **36** were evaluated against *Candida albicans*, a pathogen of major clinical importance, being one of the most common opportunistic fungi in human infections.^86^ Substances **34** and **36** inhibited their growth with MIC of 250 μg/mL, while **24** and **35** were not active (MIC > 500 μg/mL). Substances **75**, **76** and **77** were evaluated against 20 species of fungi of agricultural interest (Table S1) with MIC between 12.5 and 100 μg/mL.^15,85^ According to Ríos and Recio, 2005,^85^ the hyptolactones showed no activity against *C. albicans* and only moderate activity against the other microorganisms evaluated.

Overall, the lactones show better antibacterial than antifungal activity. This may be attributed to two factors: few antifungal assays were performed, or it may be due to their preferential interaction with bacterial targets, such as cell wall components or specific enzymes.

Since α,β-unsaturated δ-lactones, α,β,γ,δ-unsaturated δ-lactones, and α,β-unsaturated γ-lactones share the α,β-unsaturated lactone moiety and were active against these microorganisms, it indicates that this functional group is extremely important for these antimicrobial activities. Given that only α,β-unsaturated δ-lactones showed cytotoxic activity, a different behavior was observed for these activities. As reported, the selective cytotoxicity of α,β-unsaturated δ-lactones is likely due to the ideal geometric disposition and chemical reactivity of the electrophilic pharmacophore with the nucleophilic amino acid residue in α-tubulin. Antimicrobial activity, however, may depend on general interactions with membranes or enzymes that do not necessarily require the same covalent reactivity for other microbial targets, such as functional groups of fungi, like dermatophytic fungi, and protozoa.

#### 2.3.3 Antidermatophyte Activity

Substance **2** had its antifungal potential evaluated against 60 clinical strains of *Microsporum canis*, *Microsporum gypseum*, *Trichophyton mentagrophytes* and *Trichophyton rubrum*. Itraconazole was used as a control (MIC = 0.12–125 μg/mL). The substance presented similar or lower values than the control, against *M. canis* and *M. gypseum* with MIC varying between 7.8 and 32.1 μg/mL.^32^

According to Ríos and Recio, 2005,^85^ the substance presented moderate to strong antimicrobial activity, indicating that the substance can be used as antifungal agent against microorganisms responsible for dermatophytosis. The use of clinical strains, rather than solely standardized strains, enhances the relevance of the study, because clinical strains better reflects the potential clinical applicability of the substance for resistant dermatophytoses. Furthermore, it opens avenues for other hyptolactones to be evaluated

#### 2.3.4 Antiprotozoal Activity: Antichagasic, Antimalarial and Leishmanicidal

Substance **2** was evaluated for antichagasic activity (1.7%). The substances **2** and **25** were evaluated for antimalarial activity (3.4%); while, again, the substances **2**, **63** and **64** were evaluated for leishmanicidal activity (5.2%).

Substance **2** were evaluated against trypomastigote forms of *Trypanosoma cruzi*, etiological agent of Chagas disease, with IC_50_ of 940 μM,^33^ a high value compared to that of the reference drug, nifurtimox (IC_50_ = 0.72 μM),^87^ therefore, its inhibitory effect is not significant from a pharmacological standpoint. This minimal inhibition can serve as a starting point for structural modifications that could enhance potency and selectivity against the microorganism.

The substances **2** and **25** were evaluated against *Plasmodium falciparum*, parasite that causes malaria in humans. Substance **2** had an IC_50_ of 0.5 μM against *P. falciparum*,^5^ while substance **25** had an IC_50_ of 24.7 μM against *P. falciparum* sensitive to chloroquine.^9^ When compared to a reference drug, atovaquone, (IC_50_ ∼ 0.0009–0.0018 μM),^88^ substance **2** was less potent; however, it exhibits significant antimalarial potential and can be considered a promising candidate for chemical optimization and further testing. The hyptolactones **63** and **64** were evaluated against *Leishmania panamensis*, presenting IC_50_ of 9.2 and 2 μM, respectively, with better results than the control, glucantime (IC_50_ = 18.3 μM);^5^ therefore, they have potential as leishmanicidal agents.

For evaluation of hyptolactones *in vivo* study, paws and spleens of BALB/c mice infected with *Leishmania amazonensis* were treated with hyptolactone **2** (25 mg/kg). As a result, a reduction in the parasitic load was observed in the spleens by 48 and 54% when administered subcutaneously and orally (25 mg/kg), respectively, presented the same efficacy as the positive control (*N*-methylglucamine antimoniate, 100 mg/kg). Furthermore, a reducing the parasitic load in the paws in 97 and 75% was observed, after subcutaneous and oral administration, respectively,^72^ demonstrating its potential as a leishmanicidal agent. The leishmanicidal activity of substance **2** was also evaluated against *Leishmania mexicana*. At a concentration of 5.0 μg/mL, there was a marked reduction in the growth of promastigotes, while at 10 μg/mL, there was complete inhibition of their growth. According to the authors of the study, its efficiency is comparable to sodium stibogluconate, a leishmanicidal drug (concentration not reported).^61^

Although substance **2** has shown significant reduction against *Leishmania* spp., the reported studies did not specify whether the evaluated concentrations correspond to a common concentration, IC_50_, ED_50_ or another efficacy parameter. This omission limits the studies’ potential impact and introduces potential bias in the interpretation of the activity. Assuming they are common concentrations, they only provide a preliminary indication of activity but do not allow for the determination of true pharmacological potency or an adequate comparison with reference drugs. Clear specifications of the inhibitory parameter and methodological standardization are essential for a reliable evaluation against established agents.

#### 2.3.5 Inhibition of the CCR5 Receptor

The evaluation of CCR5 receptor inhibition was carried out with 3 hyptolactones plant isolates (5.2%): **10**, **11** and **12**. The chemokine receptor CCR5 is a key component of the immune system, involved in processes such as immune surveillance, inflammatory response, tumor progression, and metastasis. It plays a crucial role in attracting immune cells to sites of inflammation, guiding their migration through chemotaxis along chemokine gradients. Its endogenous ligands are chemokines, small chemoattractant cytokines involved in innate immunity that act as natural inhibitors of HIV-1 infection. These include CCL3 (MIP-1α), CCL4 (MIP-1β), CCL5 (RANTES, regulated upon activation, normal T-cell expressed and secreted), and CCL3L1. The latter is the most potent CCR5 agonist and one of the most effective suppressors of HIV-1 replication. Inhibition of CCR5 can block HIV-1 entry into host cells, as CCR5 serves as one of the coreceptors used by the virus.^89^

The inhibitory potential of substances **10**, **11** and **12** against the CCR5 chemokine receptor (U-87-CCR5 cell) through their conjugated ligands MIP-1α, MIP-1β and RANTES, was evaluated. The α,β-unsaturated δ-lactones **10**, **11** and **12** showed inhibitory activity against the MIP-1β ligand with IC_50_ of 18.6, 13.7 e 15 μM, respectively; **10** and **12** showed inhibitory activity against the MIP-1 α ligand with IC_50_ of 25.9 and 22.0 μM, respectively; and **11** showed inhibitory activity against the RANTES ligand with IC_50_ of 21.7 μM. Antiviral activity was also evaluated in a viral infection assay to determine its effect on HIV-1 infection of CCR5-positive cells. Although some antiviral activity was observed, the effects could not be distinguished from cellular cytotoxicity.^29^

The tested substances exhibited selective inhibition for the ligands. This result indicates that the hyptolactones possess specific affinity for a particular conformation or site of the CCR5 receptor. This selectivity may be biologically and therapeutically relevant, as it enables the targeted modulation of immune responses mediated by its ligands without compromising other physiological functions of the receptor. This specificity could reduce the side effects associated with the global inhibition of CCR5, suggesting potential for applications in inflammatory or infectious contexts where MIP-1β plays a critical role, including its function as a coreceptor for HIV-1 entry into host cells.

#### 2.3.6 Inhibition of the MAO-B Enzyme

The evaluation of MAO-B inhibition was carried out with 3 hyptolactones (5.2%): **72**, **73** and **74**. Monoamines are neurotransmitters containing an amine group, essential for communication between neurons and for bodily functions such as mood, movement, and sleep. Among monoamines, MAO-B is the enzyme responsible for the oxidative degradation of monoamine neurotransmitters, like dopamine. Its activity is essential for regulating dopamine levels in the central nervous system. Clinically, the selective inhibition of MAO-B is an effective therapeutic strategy for treating Parkinson’s disease, as it slows the degradation of dopamine, thereby contributing to the improvement of motor symptoms. Furthermore, this inhibition can reduce the production of reactive oxygen species, offering a potential neuroprotective effect.^90,91^

Among the substances tested, only substance **74** inhibit the MAO-B. In comparison with rasagiline (IC_50_ = 0.00443 μM),^92^ the inhibitory value of the hyptolactone **74** (IC_50_ of 15,600 μM)^30^ is practically irrelevant from a pharmacological standpoint. This finding may be important for guiding future research, indicating that focusing on α,β,γ,δ-unsaturated δ-lactones for MAO-B as a therapeutic target is not promising and that other enzymatic targets (such as MAO-A) or receptors should be investigated. However, research on MAO-B involving α,β-unsaturated δ-lactones and α,β-unsaturated γ-lactones should still be conducted.

#### 2.3.7 Antispasmodic Activity

The antispasmodic activity *ex vivo* of substance **23** was carried out in the trachea (spasms induced by carbachol) and in the ileum (spasms induced by carbachol and histamine) of *Cavia porcellus*. The substance did not exert a relaxing effect on the trachea, however, it showed activity in the ileum, inhibiting phasic contractions induced by carbachol (IC_50_ = 170 μM) and histamine (IC_50_ = 90 μM).^31^ When compared to the reference drug atropine (IC_50_ = 0.26 μM),^93^ the results indicate that the substance exhibits weak to moderate activity. Nevertheless, this compound may serve as a promising lead, opening avenues for the evaluation of other hyptolactones.

#### 2.3.8 Herbicidal Activity

Substance **3** (1000 μM) was subjected to herbicidal activity against the bud and root of *Lolium multiflorum* Lam. and *Lactuca sativa* L. This hyptolactone showed better percentages of inhibition of the growth of *L. multiflorum*, inhibiting bud and root growth by 20% and 48%, respectively.^34^

Although inhibition was observed against one of the evaluated species, the concentration can be considered high for biological assays. From an agricultural perspective, the substance lacks herbicidal potential, meaning it is not suitable for application as a weed-control agent, desiccant, or selective herbicide. From a pharmacological standpoint, however, the absence of herbicidal activity can be viewed positively, as it suggests the substance may have low toxicity and higher selectivity for future biological studies.

### 2.4 Lack of *in Vivo* Biological Evaluations of Hyptolactones

As shown in Table 2, hyptolactones were evaluated for their biological effects, mainly through *in vitro* experiments. Only one study conducted experiments on *in vivo* models.^72^ Considering that these substances have moderate to strong biological potential in certain biological activities, why are *in vivo* studies on animals scarce? One hypothesis is the fact that these substances are little known, and/or the low quantity of substances isolated from natural sources. As an alternative to the small number of substances isolated from natural products, these secondary metabolites can be obtained by organic synthesis. Unlike other secondary metabolites, with complex chemical structures, in which organic synthesis is not viable, as is the case with the synthesis of vinblastine,^94^ hyptolactones have relatively simple chemical structures. As observed in Figure 4, hyptolactones have up to five chiral carbons, a relatively low number; stereochemical control would allow selective production of specific enantiomers, which is often not feasible through isolation.

Synthesis can offer higher purity and reproducibility, avoiding seasonal or geographically limited availability, factors that are essential for pharmacological and industrial studies. The possibility of structural modification due to their simple chemical structures can generate derivatives with optimized properties, such as increased biological activity, stability, or solubility. Regarding solubility, pectinolides have an affinity for low-polarity solvents,^1,3,7,13^ so it can be assumed that the other hyptolactones have similar polarity. Orally administered drugs need to be dissolved in aqueous bodily fluids (such as gastric juice and blood) to be absorbed and transported. If a drug has low solubility in polar solvents, such as water, it will have difficulty dissolving in biological fluids, resulting in low bioavailability,^95^ which may lead to structural modification, provided the pharmacophore is preserved.

Even so, there are few scientific articles that report their syntheses and evaluate their biological effects. Given the biological potential of some hyptolactones, it is extremely important to evaluate their biological effects *in vivo* to verify their true biological potentials.

### 2.5 Toxicity

Regarding the toxic effect of hyptolactones, α,β-unsaturated δ-lactone **2** did not present toxicity against normal cells of human lung fibroblasts (MRC5),^73^ alveolar macrophage (AMJ2-C11) and murine fibroblast (BALB/c 3T3 clone A3).^28^ The α,β-unsaturated δ-lactones **58**, **60**, **61** and **62** and α,β,γ,δ-unsaturated δ-lactones **72**, **73** and **74** showed weak toxicity against *Artemia salina*.^30,40^ Ethanol extracts from the inflorescences and leaves, branches and roots of *M. pectinatum*, a species with greater amounts of isolated hyptolactones,^1,3,7,13,14,23^ did not show cytotoxicity against normal human breast cells (MCF-10A).^3^ The α,β-unsaturated δ-lactones **63** and **64** were the only substances that showed any indication of toxicity toward human pro-monocytic cells (U-937).^5^ As it can be seen, among the hyptolactones evaluated, the majority was proven to be safe, presenting no or low toxicity.

### 2.6 Risk of Bias Assessment

Among the articles added, only two analyzed the biological potential of hyptolactones in intervention studies with animals (rodents). The studies by Costa et al., 2014 ^31^ and Waechter et al., 1997 ^72^ conducted *ex vivo* and *in vivo* experiments, respectively. In each study, the substances evaluated biologically were hyptolactones **23** (antispasmodic effect) and **2** (leishmanicidal effect), respectively.

In the study by Costa et al., 2014,^31^ the reviewers JHFG and SAJ disagreed on item 8, which determined that the judgment was *yes* and *unclear*, respectively. In the study by Waechter et al., 1997,^72^ the reviewers disagreed on item 3, which determined that the judgment was *unclear* and *yes*, respectively. The results of trial (Table S2 and Table S3) and risk of bias assessment (Figure S6) of the studies can be seen in the Supporting Information.

It was observed that the studies had similar results, presenting a low risk of bias in the domains: baseline characteristics, incomplete results data and selective results reporting. The studies differed only in the other bias domain, a domain attributed by the authors of this systematic review. Among the domains adopted, it can be said that the study by Waechter et al., 1997 ^72^ is more biased than the study by Costa et al., 2014.^31^

In general, the studies presented an uncertain risk of bias in the other domains, which are specifically the domains referring to randomness during the experiment (randomization), reducing the transparency and applicability of the studies. In the clinical field, the randomized clinical trial is considered as the paradigm for evaluating the effectiveness of interventions.^96^ This type of approach is not yet a standard practice in animal experiments, which compromises the quality of reports, due to details about housing conditions or evaluation of results that are often not reported,^97,98^ especially in older studies such as the one by Waechter et al., 1997.^72^

### 2.7 Reliability of Evidence

The results of the assessment of methodological quality using Cohen’s Kappa Coefficient (k) were interpreted according to the Landis and Koch, 1977 ^99^ scale, that is, the closer to 1, the greater the agreement between reviewers. In the screening and inclusion stages of articles in the study, the value of k was 0.94 ± 0.03 (almost perfect agreement), while in the risk of bias assessment stage, the value of k was 0.83 ± 0.17 (almost perfect agreement) and 0.78 ± 0.20 (substantial agreement) for Waechter et al., 1997 ^72^ and Costa et al., 2014,^31^ respectively.

### 2.8 Final Thoughts

All substances called hyptolactones that were isolated from natural sources and underwent biological evaluations were compiled in this study.

As mentioned, the part of the molecule that increases the cellular toxicity of hyptolactones is attributed to its supposed pharmacophore group, the α,β-unsaturated δ-lactone group. However, there is no scientific evidence to prove its interactions with other biological targets.

It was observed that the lactones exhibited overall antibacterial and antifungal potential, as well as antidermatophyte, antimalarial, leishmanicidal, and possible antispasmodic activity. The hyptolactones showed no antichagasic effect or enzymatic activity against MAO-B. Regarding the activity against CCR5, the results were inconclusive, as the observed effects could not be dissociated from cellular cytotoxicity. As for the herbicidal activity, the results were negative from an agricultural perspective but positive from a pharmacological standpoint.

## 3 Conclusions

The methodological rigor in evaluating the studies present in the systematic review was fundamental for determining the 86 existing hyptolactones and their biological activities. The scientific evidence present in the 61 articles included in this study demonstrates that its biological potentials are due to the presence of α,β-unsaturated lactone in the majority of hyptolactones. The cytotoxic effect on cancer cells is the predominant activity in the class; however, only the α,β-unsaturated δ-lactones showed a cytotoxic effect, which leads us to believe that the α,β-unsaturated lactone is very important for the biological effect demonstrated. The scarcity of data regarding the *in vivo* biological potential of hyptolactones is probably a consequence of the lack of scientific knowledge about this class of substances and the small quantity that can be isolated from plants and fungi. More in-depth studies, *in vivo* models, would result in the discovery of new biological effects or even confirmation of the potential that these substances have in becoming drug candidates.

## 4 Approach

### 4.1 General Experimental Procedures

The systematic review was carried out in accordance with the PRISMA 2020 statement.^78,79^ Its development was carried out with the aim of answering the following question: “What are the hyptolactones originating from natural sources and their biological potential?” This question was formulated according to the PCC Strategy, represented by the mnemonic: population (P), concept (C) and context (C).^100^ The mnemonic assignment for the adopted question was formulated as follows: hyptolactones (population); identify the maximum number of existing hyptolactones and their biological potential (concept); originating from natural sources, isolated or synthetic substances (context).

### 4.2 Eligibility Criteria

The following inclusion criteria were adopted: articles covering all publication dates, and all languages; articles of isolation of hyptolactones with or without evidence of biological effects; and articles synthesizing hyptolactones with evidence of biological effects. The exclusion criteria were: review articles, notes, book chapters and summaries; articles synthesizing hyptolactones without evidence of biological effects; articles of isolations or syntheses of hyptolactones that presented divergences in the methodology and results of evaluating biological activities; studies of secondary metabolites that do not belong to the hyptolactone class.

### 4.3 Information Sources

The PRISMA 2020 statement establishes three steps: identification, screening and inclusion of studies.^78,79^ The study identification stage was carried out by JFGH, using electronic databases, which through preliminary research, were relevant to the study: PMC, ScienceDirect, Scopus and Web of Science. Immediately after the screening and inclusion stage (carried out by the two reviewers), the reviewers identified additional studies in the citations of the added articles and linked them to the review. The first search for scientific publications was carried out on August 20, 2020, and the last search was carried out on June 4, 2024.

### 4.4 Search Strategies

The Descritores em Ciências da Saúde (DeCS, Health Sciences Descriptors) and Medical Subject Headings (MeSH) platforms were used to search for descriptors referring to the keywords, 5,6-dihydro-α-pyrone, 5,6-dihydro-2*H*-pyran-2-one, 6-heptyl-5,6-dihydro-2*H*-pyran-2-one, δ-lactone and α-pyrone to formulate search strategies; however, no descriptors were found. In this way, search strategies were formulated using these keywords and their variations, aligned with Boolean operators (Table S4 of the Supporting Information). For the ScienceDirect, Scopus and Web of Science databases, the search for records was filtered so that the terms of the search strategies appeared in the titles, abstracts or keywords. The search was carried out without the absence of the filters adopted in the PMC, due to the small number of records found when performing this combination with the adopted search strategy or with a less limited search strategy.

### 4.5 Selection Process and Data Collection

The records extracted from the databases were added to the Rayyan online platform. First, a scan was carried out to detect and remove duplicate records (indexed in two or more databases). Then, a mapping of all remaining records was carried out in order to remove records without access. Despite being indexed in the adopted databases, some records were not accessed, even when using other databases or web pages. The evaluation of studies to be added to the systematic review was carried out in the screening and inclusion stage, by the two reviewers independently, and any disagreements were the subject of discussion.

The screening stage of the PRISMA 2020 statement, using formulated search strategy­(ies), is divided into three subscreenings.^78,79^ The first and second screening were carried out in Rayyan based on the terms of the search strategies present in the titles, abstracts or keywords, followed by the third screening, using the full text of the studies eligible for inclusion.

With the study inclusion stage completed, data extraction from the articles was carried out by the two reviewers. When analyzing the data, the reviewers reached a consensus to include new studies through citations of previously added articles. These studies were not identified by the search strategies because they did not have the chosen terms in the titles, abstracts or keywords, or because they were not indexed in these databases. Again, the identification stage was carried out by JFGH, and the screening and inclusion stages independently by the two reviewers. Subsequently, the reviewers extracted data from the articles in the new search and all the following content present in this systematic review was carried out by the two reviewers in consensus.

### 4.6 Risk of Bias Assessment

The risk of bias of articles involving the analysis of the biological potential of hyptolactones in animal intervention studies was carried out according to the SYRCLE tool.^98^ The SYRCLE tool consists of 10 signaling questions, referring to 10 domains that encompass 6 types of biases (Table S5 of the Supporting Information). For the other bias­(es) domain, it was up to the reviewers to add other types of questions that could raise concerns about the possibility of bias.

The following judgment was used as an answer for each signaling question: “yes”, indicating a low risk of bias; “uncertain”, indicating uncertain risk of bias; or “no”, indicating a high risk of bias. Thus, there was greater transparency and applicability in studies with greater numbers of *yes* answers in the judgment, indicating less biased procedures. The assessment of the risk of bias of the studies was carried out in spreadsheets on the Microsoft Excel 2019 computer program, carried out by the two reviewers independently, and disagreements were solved through discussion.

### 4.7 Certainty Assessment

To measure reliability between reviewers, in the steps carried out independently, Cohen’s Kappa Coefficient was used.^101^ Statistical analysis was performed on the GraphPad online platform.^102^

## Supplementary Material



## Notes

Product formed after the Michael addition reaction between pironetin and the cysteine residue of α-tubulin; flowchart of hyptolactones with biological effects; cytotoxic effects of the α,β-unsaturated δ-lactones; complex between substance **36** and α-tubulin; antibacterial effects of the hyptolactones against *B. subtilis*, *E. coli*, *K. aerogenes*, *M. luteus*, *P. aeruginosa*, *P. carotovorum*, *P. megaterium*, *P. vulgaris*, *S. aureus*, *S. enterica* and *S. typhi*; result of the risk of bias assessment, based on the SYRCLE tool guidelines; secondary metabolites of the hyptolactone class and their biological effects; assessment of the risk of bias in the studies based on SYRCLE tool guidelines; search strategies used to retrieve records in databases: PMC, ScienceDirect, Scopus and Web of Science; SYRCLE’s tool for assessing risk of bias (PDF)
