# Supplementary material for: Hyptolactones and Their Biological Potential: A Systematic Review
Source: J Nat Prod. 2025 Nov 25;88(12):3034–49. doi: 10.1021/acs.jnatprod.5c00965 (PMC12751116; doi:10.1021/acs.jnatprod.5c00965)
Supplement: Supplementary file 1 [file np5c00965_si_001.pdf]

Supporting Information

Hyptolactones and their biological potential: A systematic review

Felipe Gabriel Henrique Julião[1], *James Almada da Silva*[1,2*]

[1]Programa de Pós-graduação em Química, Universidade Federal de Sergipe, São Cristóvão, Sergipe, 49107-230, Brazil.
[2]Departamento de Farmácia, Universidade Federal de Sergipe, Lagarto, Sergipe, 49404-044, Brazil.
[*]Corresponding author. Email: jamesalmada@academico.ufs.br

CONTENTS

List of figures

Figure S1. General (A) and enlarged (B) views of the product formed after the Michael addition reaction between pironetin and the cysteine residue (Cys316) of α-tubulin (α2-Tub), resulting in the tubulin-RB3-TTL–pironetin complex. The chemical structure of the complex is represented using a ball-and-stick model: carbon (brown), oxygen (red), sulfur (yellow), nitrogen (blue), and hydrogen (brown, red, and blue). Illustrations adapted with permission from Yang *et al.,* 2016.[1] Copyright 2016 Springer Nature; and Wang *et al*., 2016.[2] Copyright 2016 RCSB Protein Data Bank. Licenses obtained in 2025. 4

Figure S2. Flowchart of hyptolactones with biological effects, as reported in the 61 articles included in the systematic review. 5

Figure S3. Cytotoxic effects of hyptolactones from the α,β-unsaturated δ-lactone. 30

Figure S4. Complex between pectinolide C (**36**) and α-tubulin, showing the distance between the residual amino group of the lysine amino acid (Lys352) and the β-carbon of the α,β-unsaturated lactone (H_2N-Cβ). The binding energy (E_f) values and the H_2N-Cβ bond distance are indicated. Adapted from Martínez-Fructuoso *et al.*, 2019[28], Copyright 2019 American Chemical Society......34

Figure S5. Antibacterial effects of hyptolactones. 35

Figure S6. Result of the risk of bias assessment, based on the SYRCLE tool guidelines, applied to the studies by Costa *et al*., 2014[21] and Waechter *et al*., 1997.[3] 38

List of tables

Table S1. Secondary metabolites of the hyptolactone class (originating from plants or fungi), and their biological effects#..........6

Table S2. Assessment of the risk of bias in the study by Costa *et al*., 2014,[20] based on SYRCLE tool guidelines..........30

Table S3. Assessment of the risk of bias in the study by Waechter *et al*., 1997,[2] based on SYRCLE tool guidelines..........37

Table S4. Search strategies used to retrieve records in databases..........39

Table S5. SYRCLE's tool for assessing risk of bias..........40

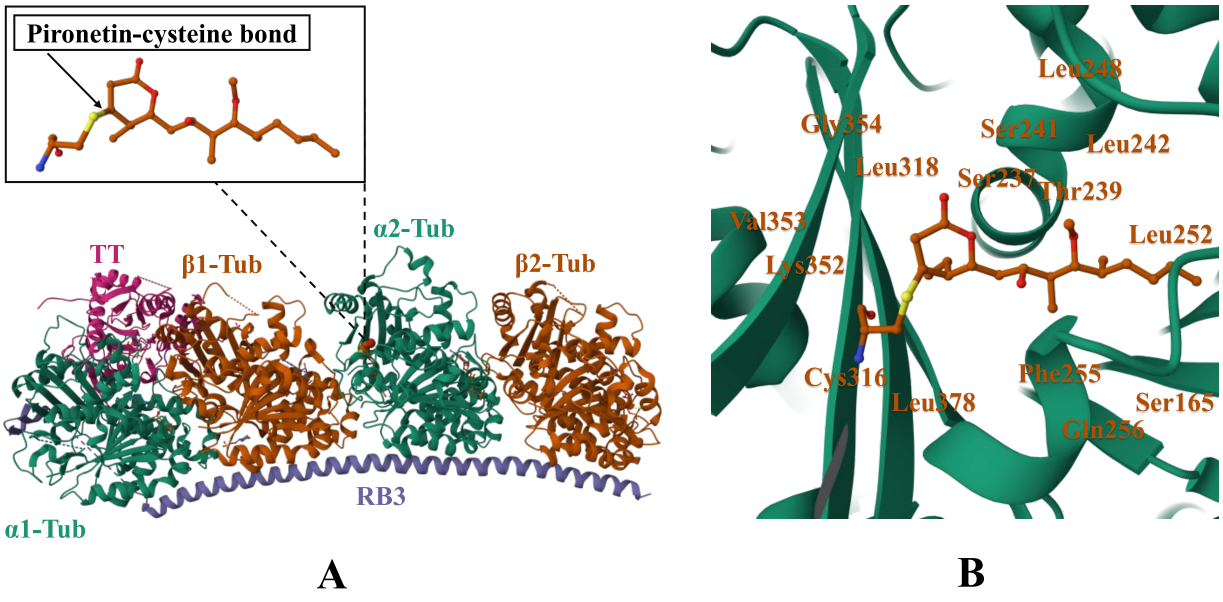

Figure S1. General (**A**) and enlarged (**B**) views of the product formed after the Michael addition reaction between pironetin and the cysteine residue (Cys316) of α-tubulin (α2-Tub), resulting in the tubulin-RB3-TTL-pironetin complex. The chemical structure of the complex is represented using a ball-and-stick model: carbon (brown), oxygen (red), sulfur (yellow), nitrogen (blue), and hydrogen (brown, red, and blue). Adapted with permission from Yang *et al.,* 2016.[1] Copyright 2016 Springer Nature. Adapted with permission from Wang *et al*., 2016.[2] Copyright 2016 RCSB Protein Data Bank.

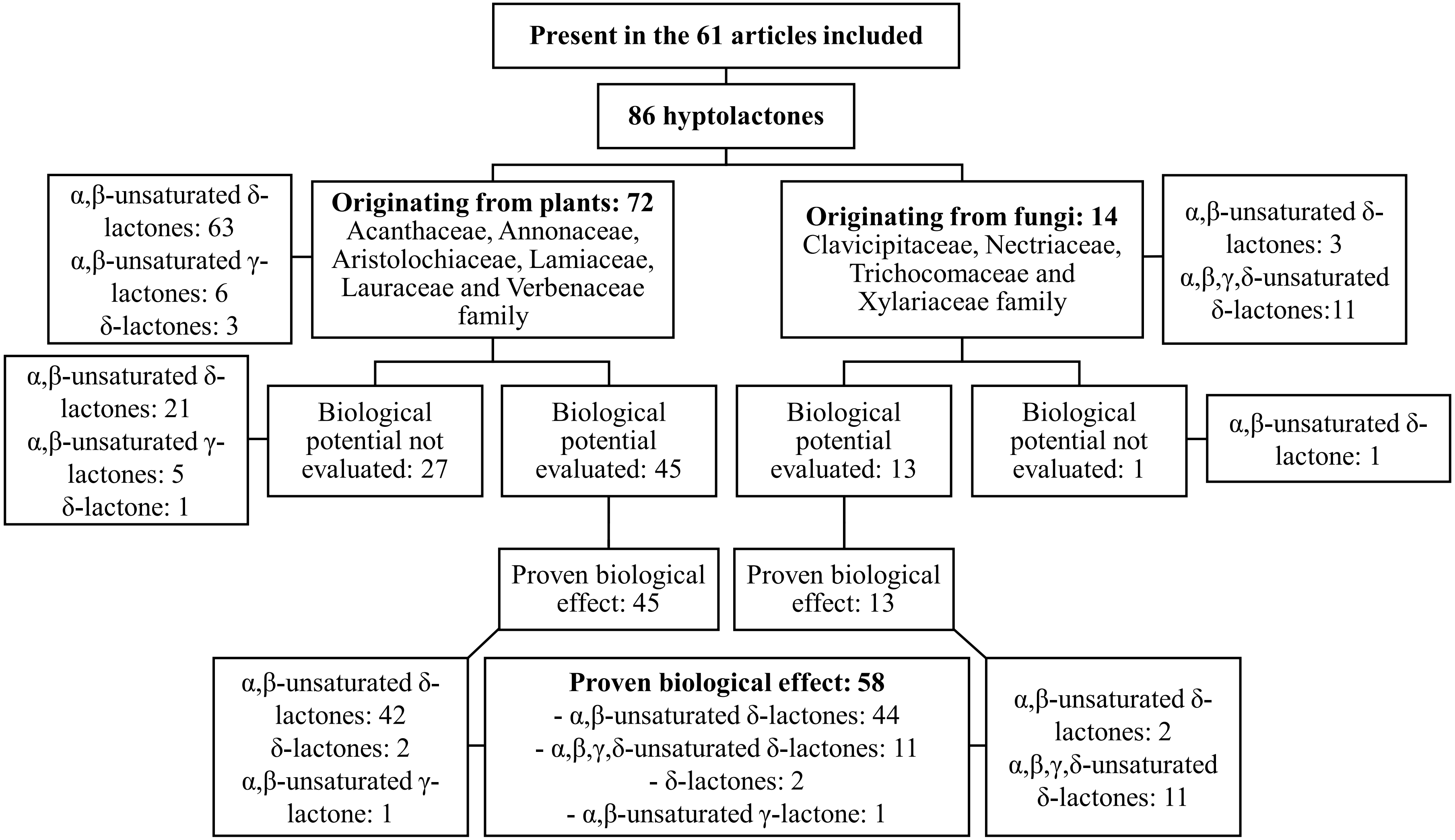

Figure S2. Flowchart of hyptolactones with biological effects, as reported in the 61 articles included in the systematic review.

Table S1. Secondary metabolites of the hyptolactone class (originating from plants or fungi), and their biological effects[#].

Hypto-lactones	Sources	Families	Biological effects	Biological models	Doses/Concentrations/ Potencies	Studies
**2**	*Annona haematantha* Miq.	Annonaceae	Leishmanicidal[α]	Paws and spleens of BALB/c infected with *Leishmania amazonensis*	25 mg/kg	Waechter *et al*., 1997[3]
	Raimondia cf. *monoica* Saff.		Antimalarial[β]	*Plasmodium falciparum*	IC_{50} = 0.5 µM	Carmona *et al*., 2003[4]
	Hyptis ovalifolia Benth.	Lamiaceae	Antidermatophyte[β]	*Microsporum canis, M. gypseum, Trichophyton rubrum* and *T. mentagrophytes.*	MIC = 7.8-125 µg/mL	de Oliveira *et al*., 2004[5]
			Antifungal[β]	*Paracoccidioides lutzii* yeasts	18 µg/mL	Prado *et al*., 2014[6]
					MIC = 72 µg/mL in the presence of acetate or glucose	
					9 µg/mL[I]	Prado *et al*., 2015[7]
						Araújo *et al*., 2016[8]
				P. lutzii, *P. brasiliensis, P. americana* and *P. restrepiensis yeasts*	MFC = 4.5-36 µg/mL	Silva *et al*., 2018[9]
				P. brasiliensis yeasts	36 µg/mL[II]	
				Silenced mutants of *P. brasiliensis* yeast	9, 18 and 36 µg/mL	Araújo *et al*., 2016[8]
			Antifungal[γ]	*P. lutzii* isocitrate lyase (PbICL)	18 µg/mL[III]	Prado *et al*., 2014[6]
				Native PbICL from yeasts	IC_{50} = 257.4 µM in the presence of acetate	
					IC_{50} = 411.8 µM in the presence of glucose	

Table S1. Secondary metabolites of the hyptolactone class (originating from plants or fungi), and their biological effects. (cont.)

Hypto-lactones	Sources	Families	Biological effects	Biological models	Doses/Concentrations/ Potencies	Studies
2	*Hyptis ovalifolia* Benth.	Lamiaceae	Antifungal[γ]	Recombinant PbICL from yeasts	IC_{50} = 28.8 µM	Prado *et al.*, 2014[6]
			Cytotoxic[β]	A549[a]	> 36 µg/mL	Silva *et al.*, 2018[9]
	Synthesized	-	Leishmanicidal[β]	*L. mexicana* promastigotes	10 µg/mL	Saeed *et al.*, 2001[10]
			Antichagasic[β]	*Trypanosoma cruzi* trypomastigotes	IC_{50} = 940 µM	de Fátima *et al.*, 2006[11]
			Cytotoxic[β]	MCF-7[a]	IC_{50} = 14.7 µM	de Fatima *et al.*, 2004[12]
				NCI-ADR[a]	IC_{50} = 11 µM	
				NCI 460[a]	IC_{50} = 14.3 µM	
				UACC62[a]	IC_{50} = 55 µM	
				786-0[a]	IC_{50} = 73.6 µM	
				OVCAR03[a]	IC_{50} = 33.8 µM	
				PC-3[a]	IC_{50} = 29 µM	
				HT-29[a]	IC_{50} = 42.3 µM	
3	Synthesized	-	Herbicide[β]	*Lolium multiflorum* Lam.	1000 µM Shoot: 20%. Root: 48%	Yamauchi *et al.*, 2012[13]
				Lactuca sativa L.	1000 µM Shoot and Root: 0%	
			Cytotoxic[β]	BC1[a]	IC_{50} = 14.4 µM	Pereda-Miranda *et al.*, 1993[14]
				HT-1080[a]	IC_{50} = 23.2 µM	
				Lu1[a]	IC_{50} = 20.1 µM	
				Mel2[a]	IC_{50} = 17 µM	
				Col2[a]	IC_{50} = 20.6 µM	

Table S1. Secondary metabolites of the hyptolactone class (originating from plants or fungi), and their biological effects. (cont.)

Hypto-lactones	Sources	Families	Biological effects	Biological models	Doses/Concentrations/Potencies	Studies
**3**	Synthesized	-	Cytotoxic[β]	KB[a]	IC_{50} = 20.6 μM	Pereda-Miranda *et al.*, 1993[14]
				KB-V[a]	IC_{50} = 44.3 μM	
				P-388[a]	IC_{50} = 5.1 μM	
				A431[a]	IC_{50} = 33.5 μM	
				LNCaP[a]	IC_{50} = 16.5 μM	
				ZR75-1[a]	IC_{50} = 44.8 μM	
**4**	*Hyptis brevipes* Poit.	Lamiaceae	Cytotoxic[β]	MCF-7	IC_{50} > 10 μM	Deng *et al.*, 2009[15]
				HT-29	IC_{50} = 5.8 μM	
**5**	*Hyptis brevipes* Poit.	Lamiaceae	Cytotoxic[β]	MCF-7	IC_{50} = 6.1 μM	Deng *et al.*, 2009[15]
				HT-29		
**6**	*Hyptis brevipes* Poit.	Lamiaceae	Cytotoxic[β]	MCF-7	IC_{50} > 10 μM	Deng *et al.*, 2009[15]
**7**	*Hyptis brevipes* Poit.	Lamiaceae	Cytotoxic[β]	MCF-7	IC_{50} > 10 μM	Deng *et al.*, 2009[15]
**8**	*Hyptis brevipes* Poit.	Lamiaceae	Cytotoxic[β]	MCF-7	IC_{50} > 10 μM	Deng *et al.*, 2009[15]
**9**	*Hyptis brevipes* Poit.	Lamiaceae	Cytotoxic[β]	MCF-7	IC_{50} = 6.7 μM	Deng *et al.*, 2009[15]
				HT-29	IC_{50} = 7.5 μM	
**10**	*Lippia alba* (Mill.) N.E.Br. ex Britton & P.Wilson	Verbenaceae	CCR5 inhibition[β,b]	MIP-1α[b]	IC_{50} = 25.9 μM	Hegde *et al.*, 2004[16]
				MIP-1β[b]	IC_{50} = 18.6 μM	
				RANTES[b]	IC_{50} > 25.9 μM	
	Hyptis brevipes Poit.	Lamiaceae	Cytotoxic[β]	MCF-7	IC_{50} = 3.6 μM	Deng *et al.*, 2009[15]
				HCT-15[a]	IC_{50} = 21.7 μM	Suárez-Ortiz *et al.*, 2013[17]

Table S1. Secondary metabolites of the hyptolactone class (originating from plants or fungi), and their biological effects. (cont.)

Hypto-lactones	Sources	Families	Biological effects	Biological models	Doses/Concentrations/ Potencies	Studies
**10**	*Hyptis brevipes* Poit.	Lamiaceae	Cytotoxic[β]	MCF-7	IC_{50} = 13.2 μM	Suárez-Ortiz *et al.*, 2013[17]
				PC-3		
				KB	IC_{50} = 0.8 μM	
				HeLa[a]	IC_{50} = 0.2 μM	
				Hep-2[a]	IC_{50} = 8.8 μM	
**11**	*Lippia alba* (Mill.) N.E.Br. ex Britton & P.Wilson	Verbenaceae	CCR5 inhibition[β]	MIP-1α	IC_{50} > 25.0 μM	Hegde *et al.*, 2004[16]
				MIP-1β	IC_{50} = 13.7 μM	
				RANTES	IC_{50} = 21.7 μM	
	Hyptis brevipes Poit.	Lamiaceae	Cytotoxic[β]	HCT-15	IC_{50} = 17 μM	Suárez-Ortiz *et al.*, 2013[17]
				MCF-7	IC_{50} = 13 μM	
				KB	IC_{50} = 5.0 μM	
				PC-3	IC_{50} = 13.5 μM	
				HeLa	IC_{50} = 14.5 μM	
				Hep-2	IC_{50} = 20.7 μM	
**12**	*Lippia alba* (Mill.) N.E.Br. ex Britton & P.Wilson	Verbenaceae	CCR5 inhibition[β]	MIP-1α	IC_{50} = 22.0 μM	Hegde *et al.*, 2004[16]
				MIP-1β	IC_{50} = 15.0 μM	
				RANTES	IC_{50} > 25.0 μM	
	Hyptis brevipes Poit.	Lamiaceae	Cytotoxic[β]	HCT-15	IC_{50} = 14 μM	Suárez-Ortiz *et al.*, 2013[17]
				MCF-7	IC_{50} = 17.7 μM	
				KB	IC_{50} = 4.7 μM	

Table S1. Secondary metabolites of the hyptolactone class (originating from plants or fungi), and their biological effects. (cont.)

Hypto-lactones	Sources	Families	Biological effects	Biological models	Doses/Concentrations/ Potencies	Studies
**12**	*Hyptis brevipes* Poit.	Lamiaceae	Cytotoxic[β]	PC-3	IC_{50} = 11.7 μM	Suárez-Ortiz *et al.*, 2013[17]
				HeLa	IC_{50} = 11 μM	
				Hep-2	IC_{50} = 15.7 μM	
**13**	*Hyptis brevipes* Poit.	Lamiaceae	Cytotoxic[β]	HCT-15	IC_{50} = 25.7 μM	9
				MCF-7	IC_{50} = 8.4 μM	
				KB	IC_{50} = 2.9 μM	
				PC-3	IC_{50} = 13.9 μM	
				HeLa	IC_{50} = 5.5 μM	
				Hep-2	IC_{50} = 14.6 μM	
**14**	*Hyptis brevipes* Poit.	Lamiaceae	Cytotoxic[β]	HCT-15	IC_{50} > 10 μM	Suárez-Ortiz *et al.*, 2017[18]
				MCF-7		
				Hep-2		
				KB	IC_{50} = 9.5 μM	
				PC-3	IC_{50} = 10 μM	
				HeLa	IC_{50} = 4.2 μM	
**15**	*Hyptis brevipes* Poit.	Lamiaceae	Cytotoxic[β]	HCT-15	IC_{50} > 10 μM	Suárez-Ortiz *et al.*, 2017[18]
				MCF-7	IC_{50} = 9.3 μM	
				KB	IC_{50} = 7.5 μM	
				PC-3	IC_{50} = 8.8 μM	
				HeLa	IC_{50} = 3.3 μM	

Table S1. Secondary metabolites of the hyptolactone class (originating from plants or fungi), and their biological effects. (cont.)

Hypto-lactones	Sources	Families	Biological effects	Biological models	Doses/Concentrations/ Potencies	Studies
**15**	*Hyptis brevipes* Poit.	Lamiaceae	Cytotoxic$^{\beta}$	Hep-2	IC_{50} = 7.7 μM	Suárez-Ortiz *et al.*, 2017[18]
**16**	*Hyptis brevipes* Poit.	Lamiaceae	Cytotoxic$^{\beta}$	HCT-15	IC_{50} > 10 μM	Suárez-Ortiz *et al.*, 2017[18]
				MCF-7		
				KB	IC_{50} = 1.7 μM	
				PC-3	IC_{50} = 7.5 μM	
				HeLa	IC_{50} = 6.0 μM	
				Hep-2	IC_{50} = 5.7 μM	
**17**	*Hyptis brevipes* Poit.	Lamiaceae	Cytotoxic$^{\beta}$	HCT-15	IC_{50} > 10 μM	Suárez-Ortiz *et al.*, 2017[18]
				MCF-7		
				PC-3		
				Hep-2		
				KB	IC_{50} = 3.6 μM	
				HeLa	IC_{50} = 9.5 μM	
**18**	*Hyptis brevipes* Poit.	Lamiaceae	Cytotoxic$^{\beta}$	HCT-15	IC_{50} > 10 μM	Suárez-Ortiz *et al.*, 2017[18]
				MCF-7		
				PC-3		
				KB	IC_{50} = 4.9 μM	
				HeLa	IC_{50} = 6.9 μM	
				Hep-2	IC_{50} = 9.4 μM	
**20**	Synthesized	-	Cytotoxic$^{\beta}$	HeLa	IC_{50} = 3.5 μM	Sabitha *et al.*, 2012[19]

Table S1. Secondary metabolites of the hyptolactone class (originating from plants or fungi), and their biological effects. (cont.)

Hypto-lactones	Sources	Families	Biological effects	Biological models	Doses/Concentrations/ Potencies	Studies
**20**	Synthesized	-	Cytotoxic[β]	MCF-7	IC_{50} = 8.2 µM	Sabitha *et al.*, 2012[19]
				A549	IC_{50} = 1.5 µM	
**21***	*Epichloë typhina*	Clavicipitaceae	Antifungal[β]	*Cladosporium herbarum*	25 µg/spot[IV]	Koshino *et al.*, 1992[20]
**23**	*Hyptis macrostachys* Benth.	Lamiaceae	Antispasmodic[δ]	Spasms in the ileum of *Cavia porcellus*	Histamine induced: IC_{50} = 90 µM	Costa *et al.*, 2014[21]
					Carbachol induced: IC_{50} = 170 µM	
				Spasms in the trachea of *C. porcellus*	Carbachol induced: > 100 µM	
**24**	*Mesosphaerum pectinatum* (L.) Kuntze	Lamiaceae	Antifungal[β]	*Candida albicans*	MIC > 500 µg/mL	Pereda-Miranda *et al.*, 1993[14]
			Antibacterial[β]	*Staphylococcus aureus*	MIC > 100 µg/mL	
				Escherichia coli	MIC > 500 µg/mL	
				Pseudomonas aeruginosa		
				Bacillus subtilis	MIC = 100 µg/mL	
						Rojas *et al.*, 1992[22]
					Extremely sensitive[V]	Bambang *et al.*, 2020[23]
				S. aureus	Relatively sensitive	
				Streptococcus mutans		
				Salmonella Typhi		
				E. coli	Moderately sensitive	
			Cytotoxic[β]	MCF-7	IC_{50} = 208.5 µM	Suzery *et al.*, 2020[24]
				T47D[a]	IC_{50} = 493 µM	

Table S1. Secondary metabolites of the hyptolactone class (originating from plants or fungi), and their biological effects. (cont.)

Hypto-lactones	Sources	Families	Biological effects	Biological models	Doses/Concentrations/ Potencies	Studies
**24**	*Mesosphaerum pectinatum* (L.) Kuntze	Lamiaceae	Cytotoxic[β]	MDA-MB-231[a]	IC_{50} = 15.2 μM	Santana *et al.*, 2019[25]
				BC1	IC_{50} = 6.5 μM	Pereda-Miranda *et al.*, 1993[14]
				HT-1080	IC_{50} = 26.1 μM	
				Lu1	IC_{50} = 13.3 μM	
				Mel2	IC_{50} = 33.1 μM	
				Col2	IC_{50} = 12.8 μM	
				ZR75-1	IC_{50} = 17.4 μM	
				LNCaP	IC_{50} = 20.4 μM	
				P-388	IC_{50} = 4.3 μM	
				A431	IC_{50} = 10 μM	
				KB-V	IC_{50} = 12.2 μM	
				KB	IC_{50} = 9.8 μM	
	Not informed	-		Hep-2	IC_{50} = 16.8 μM	Mendoza-Espinoza *et al.*, 2009[26]
				HeLa	IC_{50} = 7.6 μM	
**25**	*Lippia javanica* (Burm.f.) Spreng.	Verbenaceae	Antimalarial[β]	*P. falciparum* sensitive to chloroquine	IC_{50} = 24.7 μM	Ludere *et al.*, 2013[27]
**26**	*Hyptis monticola* Mart. ex Benth.	Lamiaceae	Cytotoxic[γ]	MCF-7	IC_{50} = 7.7 μM	Martínez-Fructuoso *et al.*, 2019[28]
				HeLa	IC_{50} = 2.7 μM	
				HCT-15	IC_{50} >10 μM	
**27**	*Hyptis monticola* Mart. ex Benth.	Lamiaceae	Cytotoxic[γ]	MCF-7	IC_{50} = 9.8 μM	Martínez-Fructuoso *et al.*, 2019[28]

Table S1. Secondary metabolites of the hyptolactone class (originating from plants or fungi), and their biological effects. (cont.)

Hypto-lactones	Sources	Families	Biological effects	Biological models	Doses/Concentrations/ Potencies	Studies
**27**	*Hyptis monticola* Mart. ex Benth.	Lamiaceae	Cytotoxic[γ]	HCT-15	IC_{50} = 9.9 μM	Martínez-Fructuoso *et al.*, 2019[28]
				HeLa	IC_{50} = 6.9 μM	
**32**	*Neohyptis paniculata* (Baker) J.K.Morton	Lamiaceae	Antibacterial[β]	MDRSA[c]	MIC = 64 μg/mL	Rahman and Gibbons, 2015[29]
				MRSA[c]		
				S. aureus	MIC = 128 μg/mL	
**34**	*Mesosphaerum pectinatum* (L.) Kuntze	Lamiaceae	Antifungal[β]	*C. albicans*	MIC = 250 μg/mL	Pereda-Miranda *et al.*, 1993[14]
			Antibacterial[β]	*B. subtilis*	MIC = 6.3 μg/mL	
				S. aureus	MIC = 12.5 μg/mL	
				E. coli	MIC = 200 μg/mL	
				P. aeruginosa		
				S. aureus	MIC = 32 μg/mL	Fragoso-Serrano *et al.*, 2005[30]
				MDRSA	MIC = 128 μg/mL	
				MRSA		
			Cytotoxic[β]	BC1	IC_{50} = 3.2 μM	Pereda-Miranda *et al.*, 1993[14]
				Col2		
				HT-1080	IC_{50} = 5.5 μM	
				Lu1	IC_{50} = 2.9 μM	
				Mel2	IC_{50} = 2.3 μM	
				KB	IC_{50} = 5.8 μM	
					IC_{50} = 1.9 μM	Fragoso-Serrano *et al.*, 2005[30]

Table S1. Secondary metabolites of the hyptolactone class (originating from plants or fungi), and their biological effects. (cont.)

Hypto-lactones	Sources	Families	Biological effects	Biological models	Doses/Concentrations/ Potencies	Studies
**34**	*Mesosphaerum pectinatum* (L.) Kuntze	Lamiaceae	Cytotoxic[β]	KB-V	IC_{50} = 5.8 μM	Pereda-Miranda *et al*., 1993[14]
				P-388	IC_{50} = 2.9 μM	
				A431	IC_{50} = 4.5 μM	
				LNCaP	IC_{50} = 2.3 μM	
				ZR75-1	IC_{50} = 11.6 μM	
				MCF-7	IC_{50} = 3.2 μM	Martínez-Fructuoso *et al*., 2019[28]
				HeLa		
				HCT-15	IC_{50} = 5.7 μM	
**35**	*Mesosphaerum pectinatum* (L.) Kuntze	Lamiaceae	Antifungal[β]	*C. albicans*	MIC = 300 μg/mL	Pereda-Miranda *et al*., 1993[14]
			Antibacterial[β]	*B. subtilis*	MIC = 25 μg/mL	
				S. aureus	MIC = 100 μg/mL	
				E. coli	MIC = 300 μg/mL	
				P. aeruginosa		
				S. aureus	MIC = 128 μg/mL	Fragoso-Serrano *et al*., 2005[30]
				MDRSA	MIC = 256 μg/mL	
				MRSA		
			Cytotoxic[β]	BC1	IC_{50} = 9.3 μM	Pereda-Miranda *et al*., 1993[14]
				HT-1080	IC_{50} = 8.6 μM	
				Lu1	IC_{50} = 14.2 μM	
				Mel2	IC_{50} = 8.2 μM	

Table S1. Secondary metabolites of the hyptolactone class (originating from plants or fungi), and their biological effects. (cont.)

Hypto-lactones	Sources	Families	Biological effects	Biological models	Doses/Concentrations/ Potencies	Studies
**35**	*Mesosphaerum pectinatum* (L.) Kuntze	Lamiaceae	Cytotoxic[β]	Col2	IC_{50} = 4.1 μM	Pereda-Miranda *et al.*, 1993[14]
				KB	IC_{50} = 5.2 μM	
					IC_{50} > 74.5 μM	Fragoso-Serrano *et al.*, 2005[30]
				KB-V	IC_{50} = 7.5 μM	Pereda-Miranda *et al.*, 1993[14]
				P-388	IC_{50} = 0.4 μM	
				A431	IC_{50} = 2.2 μM	
				LNCaP	IC_{50} = 3.4 μM	
				ZR75-1	IC_{50} = 6.0 μM	
				MCF-7	IC_{50} = 9.3 μM	Martínez-Fructuoso *et al.*, 2019[28]
				HeLa	IC_{50} = 4.1 μM	
				HCT-15	IC_{50} = 5.2 μM	
**36**	*Mesosphaerum pectinatum* (L.) Kuntze	Lamiaceae	Antifungal[β]	*C. albicans*	MIC > 500 μg/mL	Pereda-Miranda *et al.*, 1993[14]
			Antibacterial[β]	*B. subtilis*	MIC = 12.5 μg/mL	
				S. aureus	MIC = 100 μg/mL	
				E. coli	MIC > 500 μg/mL	
				P. aeruginosa		
				S. aureus	MIC = 64 μg/mL	Fragoso-Serrano *et al.*, 2005[30]
				MDRSA	MIC = 128 and 256 μg/mL[VI]	
				MRSA	MIC = 128 μg/mL	

Table S1. Secondary metabolites of the hyptolactone class (originating from plants or fungi), and their biological effects. (cont.)

Hypto-lactones	Sources	Families	Biological effects	Biological models	Doses/Concentrations/ Potencies	Studies
**36**	*Mesosphaerum pectinatum* (L.) Kuntze	Lamiaceae	Cytotoxic$^{\beta}$	BC1	IC_{50} = 7.5 µM	Pereda-Miranda *et al.*, 1993[14]
				HT-1080	IC_{50} = 6.7 µM	
				Lu1	IC_{50} = 8.6 µM	
				Mel2	IC_{50} = 12.3 µM	
				Col2	IC_{50} = 6.0 µM	
				KB	IC_{50} = 6.3 µM	
					IC_{50} = 9.3 µM	Fragoso-Serrano *et al.*, 2005[30]
				KB-V	IC_{50} = 11.9 µM	Pereda-Miranda *et al.*, 1993[14]
				P-388	IC_{50} = 8.2 µM	
				A431	IC_{50} = 3.0 µM	
				LNCaP		
				ZR75-1	IC_{50} = 7.1 µM	
			Cytotoxic$^{\gamma}$	MCF-7	IC_{50} = 1.0 µM	Martínez-Fructuoso *et al.*, 2019[28]
				HeLa	IC_{50} = 0.3 µM	
				HCT-15	IC_{50} = 2.3 µM	
**38**	*Mesosphaerum pectinatum* (L.) Kuntze	Lamiaceae	Cytotoxic$^{\beta}$	MDA-MB-231	IC_{50} = 66.8 µM	Santana *et al.*, 2019[25]
**39**	*Mesosphaerum pectinatum* (L.) Kuntze	Lamiaceae	Cytotoxic$^{\beta}$	MCF-7	IC_{50} = 5.9 µM	Martínez-Fructuoso *et al.*, 2019[28]
				HeLa	IC_{50} = 6.2 µM	
				HCT-15	IC_{50} = 7.4 µM	

Table S1. Secondary metabolites of the hyptolactone class (originating from plants or fungi), and their biological effects. (cont.)

Hypto-lactones	Sources	Families	Biological effects	Biological models	Doses/Concentrations/ Potencies	Studies
**40**	*Mesosphaerum pectinatum* (L.) Kuntze	Lamiaceae	Cytotoxic[β]	MCF-7	IC_{50} = 1.3 µM	Martínez-Fructuoso *et al.*, 2019[28]
				HeLa	IC_{50} = 1.4 µM	
				HCT-15	IC_{50} = 9.5 µM	
**41**	*Mesosphaerum pectinatum* (L.) Kuntze	Lamiaceae	Cytotoxic[γ]	MCF-7	IC_{50} = 0.5 µM	Martínez-Fructuoso *et al.*, 2019[28]
				HeLa	IC_{50} = 0.7 µM	
				HCT-15	IC_{50} = 0.8 µM	
**42**	*Mesosphaerum pectinatum* (L.) Kuntze	Lamiaceae	Cytotoxic[β]	MDA-MB-231	IC_{50} = 39.5 µM	Santana *et al.*, 2019[25]
**44**	*Hyptis spicigera* Lam.	Lamiaceae	Cytotoxic[β]	KB	IC_{50} = 3.5 µM	Pereda-Miranda *et al.*, 2001[31]
	Synthesized	-		HCT-15	IC_{50} = 40.8 µM	Falomir *et al.*, 2003[32]
				SQC-1 UISO[a]	IC_{50} = 37.3 µM	
				KB	IC_{50} = 13.6 µM	
				OVCAR[a]	IC_{50} = 44.6 µM	
	Not informed			Hep-2	IC_{50} = 22 µM	Mendoza-Espinoza *et al.*, 2009[26]
				KB	IC_{50} = 12.7 µM	
				HeLa	IC_{50} = 37.3 µM	
**54**	Synthesized	-	Cytotoxic[β]	HeLa	GI_{50} = 7.4 µM	Sabitha *et al.*, 2014[33]
				PANC 1[a]	GI_{50} = 0.1 µM	
				HepG2[a]	GI_{50} = 5.7 µM	
				SK-N-SH[a]	GI_{50} = 10.2 µM	

Table S1. Secondary metabolites of the hyptolactone class (originating from plants or fungi), and their biological effects. (cont.)

Hypto-lactones	Sources	Families	Biological effects	Biological models	Doses/Concentrations/ Potencies	Studies
**55**	Synthesized	-	Cytotoxic[β]	HeLa	IC_{50} = 0.2 µM	Sabitha *et al.*, 2012[19]
				MDA-MB-231	IC_{50} = 0.6 µM	
				MCF-7	IC_{50} = 1.9 µM	
				A549	IC_{50} = 0.9 µM	
**56***	*Xylariales* sp.	Xylariaceae	Antibacterial[β]	*Pectobacterium carotovorum*	MIC = 20.5 µg/mL	Guo *et al.*, 2018[34]
			Cytotoxic[β]	A549	IC_{50} > 208.1 µM	
				HepG2		
				CaSki[a]		
				MDA-MB-231		
**58**	*Neohyptis paniculata* (Baker) J.K.Morton	Lamiaceae	Antibacterial[β]	MRSA	MIC = 64 and 128 µg/mL[VI]	Rahman and Gibbons, 2015[29]
				S. aureus	MIC = 64 and 128 µg/mL	
	Hyptis oblongifolia Benth.			*B. subtilis*	MIC = 100 µg/mL	Rojas *et al.*, 1992[22]
			Cytotoxic[β]	A549	IC_{50} = 230.6 µM	Pereda-Miranda *et al.*, 1990[35]
				HT-29	IC_{50} = 124.3 µM	
				MCF-7	IC_{50} = 113.5 µM	
	Synthesized	-		BC1	IC_{50} = 14.8 µM	Pereda-Miranda *et al.*, 1993[14]
				HT-1080	IC_{50} = 17.6 µM	
				Lu1	IC_{50} = 10.2 µM	
				Mel2	IC_{50} = 12.6 µM	

Table S1. Secondary metabolites of the hyptolactone class (originating from plants or fungi), and their biological effects. (cont.)

Hypto-lactones	Sources	Families	Biological effects	Biological models	Doses/Concentrations/ Potencies	Studies
**58**	Synthesized	-	Cytotoxic[β]	Col2	IC_{50} = 9.2 μM	Pereda-Miranda *et al.*, 1993[14]
				KB	IC_{50} = 8.9 μM	
				KB-V	IC_{50} = 10.5 μM	
				P-388	IC_{50} = 1.2 μM	
				A431	IC_{50} = 5.9 μM	
				LNCaP	IC_{50} = 5.5 μM	
				ZR75-1	IC_{50} = 14.5 μM	
**59**	*Rabdosia ternifolia* (D.Don) H.Hara	Lamiaceae	Cytotoxic[β]	BC1	IC_{50} = 12.3 μM	Lu *et al.*, 1997[36]
				KB		
				Lu1	IC_{50} = 33 μM	
				Col2	IC_{50} = 14.4 μM	
				KB-V (-VLB)[a]		
				LNCaP	IC_{50} = 3.1 μM	
				KB-V (+VLB)[a]	IC_{50} = 5.8 μM	
**60**	*Hyptis oblongifolia* Benth.	Lamiaceae	Antibacterial[β]	*B. subtilis*	MIC = 100 μg/mL	Rojas *et al.*, 1992[22]
**61**	*Hyptis oblongifolia* Benth.	Lamiaceae	Antibacterial[β]	*B. subtilis*	MIC = 100 μg/mL	Rojas *et al.*, 1992[22]
**62**	*Hyptis oblongifolia* Benth.	Lamiaceae	Antibacterial[β]	*B. subtilis*	MIC = 100 μg/mL	Rojas *et al.*, 1992[22]
			Cytotoxic[β]	A549	IC_{50} = 132 μM	Pereda-Miranda *et al.*, 1990[35]
				HT-29	IC_{50} = 81.4 μM	
				MCF-7	IC_{50} = 73.2 μM	

Table S1. Secondary metabolites of the hyptolactone class (originating from plants or fungi), and their biological effects. (cont.)

Hypto-lactones	Sources	Families	Biological effects	Biological models	Doses/Concentrations/ Potencies	Studies
**63**	*Raimondia* cf. *monoica* Saff.	Annonaceae	Leishmanicidal[β]	*L. panamensis* promastigotes	IC_{50} = 9.2 µg/mL	Carmona *et al.*, 2003[4]
**64**	*Raimondia* cf. *monoica* Saff.	Annonaceae	Leishmanicidal[β]	*L. panamensis* promastigotes	IC_{50} = 2.0 µg/mL	Carmona *et al.*, 2003[4]
**67***	*Fusarium* sp. F20	Nectriaceae	Antibacterial[β]	*B. subtilis*	> 1000 µg/mL	Gao *et al.*, 2020[37]
				S. aureus		
				E. coli		
				P. aeruginosa		
				Chromobacterium violaceum[d]		
**68***	*Fusarium* sp. F20	Nectriaceae	Antibacterial[β]	*B. subtilis*	> 1000 µg/mL	Gao *et al.*, 2020[37]
				S. aureus		
				E. coli		
				P. aeruginosa		
				C. violaceum[d]		
**69***	*Xylariales* sp.	Xylariaceae	Antibacterial[β]	*P. carotovorum*	MIC = 25.5 µg/mL	Guo *et al.*, 2018[34]
			Cytotoxic[β]	A549	IC_{50} > 209.8 µM	
				HepG2		
				CaSki		
				MDA-MB-231		
**70***	*Xylariales* sp.	Xylariaceae	Antibacterial[β]	*P. carotovorum*	MIC = 22.6 µg/mL	Guo *et al.*, 2018[34]
			Cytotoxic[β]	A549	IC_{50} > 186.4 µM	

Table S1. Secondary metabolites of the hyptolactone class (originating from plants or fungi), and their biological effects. (cont.)

Hypto-lactones	Sources	Families	Biological effects	Biological models	Doses/Concentrations/ Potencies	Studies
**70***	*Xylariales* sp.	Xylariaceae	Cytotoxic[β]	HepG2	IC_{50} > 186.4 µM	Guo *et al.*, 2018[34]
				CaSki		
				MDA-MB-231		
**71***	*Xylariales* sp.	Xylariaceae	Antibacterial[β]	*P. carotovorum*	MIC = 24.7 µg/mL	Guo *et al.*, 2018[34]
			Cytotoxic[β]	A549	IC_{50} > 186.4 µM	
				HepG2		
				CaSki		
				MDA-MB-231		
**72***	*Xylariales* sp.	Xylariaceae	Enzyme inhibition[β]	MAO-B	IC_{50} > 100,000 µM	Yang *et al.*, 2020[38]
			Antibacterial[β]	*E. coli*	MIC = 25.6 µg/mL	
				S. aureus	MIC = 46.9 µg/mL	
				P. aeruginosa	MIC = 55.6 µg/mL	
				P. carotovorum	MIC > 100 µg/mL	
**73***	*Xylariales* sp.	Xylariaceae	Enzyme inhibition[β]	MAO-B	IC_{50} > 100,000 µM	Yang *et al.*, 2020[38]
			Antibacterial[β]	*E. coli*	MIC = 32.4 µg/mL	
				S. aureus	MIC = 47.8 µg/mL	
				P. aeruginosa	MIC = 57.9 µg/mL	
				P. carotovorum	MIC > 100 µg/mL	
**74***	*Xylariales* sp.	Xylariaceae	Enzyme inhibition[β]	MAO-B	IC_{50} = 15,600 µM	Yang *et al.*, 2020[38]
			Antibacterial[β]	*E. coli*	MIC = 57.8 µg/mL	

Table S1. Secondary metabolites of the hyptolactone class (originating from plants or fungi), and their biological effects. (cont.)

Hypto-lactones	Sources	Families	Biological effects	Biological models	Doses/Concentrations/ Potencies	Studies
**74***	*Xylariales* sp.	Xylariaceae	Antibacterial[β]	*S. aureus*	MIC = 48.9 µg/mL	Yang *et al*., 2020[38]
				P. aeruginosa	MIC = 56.3 µg/mL	
				P. carotovorum	MIC > 100 µg/mL	
**75***	*Penicillium ochrochloron*	Trichocomaceae	Antifungal[β]	*Cercospora arachidicola*	MIC = 12.5 µg/mL	Zhao *et al*., 2018[39]
				Alternaria solani		
				Fusarium graminearum		
				Bipolaris carbonum	MIC = 25 µg/mL	
				Sclerotinia sclerotiorum		
				Cylindrocladium parasiticum		
				A. alternata f. sp. *mali*		
				C. personata		
				Botrytis cinerea		
				Ustilago scitaminea		
				Colletotrichum gloeosporioides		
				Rhizoctonia cerealis	MIC = 50 µg/mL	
				Helminthosporium maydis		
				C. orbiculare		
				Ascochyta gossypii		
				A. alternata		

Table S1. Secondary metabolites of the hyptolactone class (originating from plants or fungi), and their biological effects. (cont.)

Hypto-lactones	Sources	Families	Biological effects	Biological models	Doses/Concentrations/ Potencies	Studies
**75***	*Penicillium ochrochloron*	Trichocomaceae	Antifungal[β]	*B. fabiopsis*	MIC = 50 µg/mL	Zhao *et al*., 2018[39]
				A. brassicae		
				C. graminicola	MIC = 100 µg/mL	
				Exserohilum turcicum		
			Antibacterial[β]	*B. subtilis*	MIC = 50 µg/mL	
				Micrococcus luteus		
				S. aureus		
				Priestia megaterium		
				Salmonella enterica		
				Proteus vulgaris		
				S. Typhi		
				P. aeruginosa		
				E. coli		
				Klebsiella aerogenes		
			Cytotoxic[β]	A549	IC_{50} > 423.2 µM	
				Huh7[a]		
				LN229[a]		
				MGC[a]		
				MHCC97H[a]		
				LOVO[a]		

Table S1. Secondary metabolites of the hyptolactone class (originating from plants or fungi), and their biological effects. (cont.)

Hypto-lactones	Sources	Families	Biological effects	Biological models	Doses/Concentrations/ Potencies	Studies
**75***	*Penicillium ochrochloron*	Trichocomaceae	Cytotoxic[β]	MDA-MB-231[a]	IC_{50} > 423.2 µM	Zhao *et al*., 2018[39]
**76***	*Penicillium ochrochloron*	Trichocomaceae	Antifungal[β]	*C. arachidicola*	MIC = 12.5 µg/mL	Zhao *et al*., 2018[39]
				F. graminearum		
				A. solani	MIC = 25 µg/mL	
				B. carbonum		
				C.personata		
				C. parasiticum		
				U. scitaminea		
				C. graminicola		
				A. alternata f. sp. *mali*	MIC = 50 µg/mL	
				B. fabiopsis		
				B. cinerea		
				A. alternata (Fries) Keissler		
				R. cerealis		
				H. maydis		
				C. orbiculare		
				A. gossypii		
				S. sclerotiorum	MIC = 100 µg/mL	
				C. gloeosporioides		
				A. brassicae	MIC > 100 µg/mL	

Table S1. Secondary metabolites of the hyptolactone class (originating from plants or fungi), and their biological effects. (cont.)

Hypto-lactones	Sources	Families	Biological effects	Biological models	Doses/Concentrations/ Potencies	Studies
**76***	*Penicillium ochrochloron*	Trichocomaceae	Antifungal[β]	*E. turcicum*	MIC > 100 μg/mL	Zhao *et al.*, 2018[39]
			Antibacterial[β]	*S.* Typhi	MIC = 25 μg/mL	
				B. subtilis	MIC = 50 μg/mL	
				M. luteus		
				S. aureus		
				P. megaterium		
				S. enterica		
				P. vulgaris		
				P. aeruginosa		
				E. coli		
				K. aerogenes	MIC = 100 μg/mL	
			Cytotoxic[β]	A549	IC_{50} > 419.7 μM	
				Huh7		
				LN229		
				MGC		
				MHCC97H		
				LOVO		
				MDA-MB-231		
**77***	*Penicillium ochrochloron*	Trichocomaceae	Antifungal[β]	*A. solani*	MIC = 12.5 μg/mL	Zhao *et al.*, 2018[39]
				C. parasiticum		

Table S1. Secondary metabolites of the hyptolactone class (originating from plants or fungi), and their biological effects. (cont.)

Hypto-lactones	Sources	Families	Biological effects	Biological models	Doses/Concentrations/ Potencies	Studies
**77***	*Penicillium ochrochloron*	Trichocomaceae	Antifungal[β]	*A. alternata* f. sp. *mali*	MIC = 12.5 µg/mL	Zhao *et al.*, 2018[39]
				C. arachidicola	MIC = 25 µg/mL	
				B. carbonum		
				B. cinerea		
				C. graminicola		
				R. cerealis		
				F. graminearum	MIC = 50 µg/mL	
				C.personata		
				U. scitaminea		
				H. maydis		
				C. orbiculare		
				A. gossypii		
				A. alternata (Fries) Keissler		
				C. gloeosporioides		
				B. fabiopsis	MIC = 100 µg/mL	
				A. brassicae	MIC > 100 µg/mL	
				S. sclerotiorum		
				E. turcicum		
			Antibacterial[β]	*B. subtilis*	MIC = 50 µg/mL	
				M. luteus		

Table S1. Secondary metabolites of the hyptolactone class (originating from plants or fungi), and their biological effects. (cont.)

Hypto-lactones	Sources	Families	Biological effects	Biological models	Doses/Concentrations/ Potencies	Studies
**77***	*Penicillium ochrochloron*	Trichocomaceae	Antibacterial[β]	*S. aureus*	MIC = 50 μg/mL	Zhao *et al*., 2018[39]
				P. megaterium		
				S. enterica		
				P. vulgaris		
				S. Typhi		
				P. aeruginosa		
				E. coli		
				K. aerogenes		
			Cytotoxic[β]	A549	IC_{50} > 416.1 μM	
				Huh7		
				LN229		
				MGC		
				MHCC97H		
				LOVO		
				MDA-MB-231		
**80**	*Mesosphaerum pectinatum* (L.) Kuntze	Lamiaceae	Antibacterial[β]	*S. aureus*	MIC = 32 μg/mL	Fragoso-Serrano *et al*., 2005[30]
				MDRSA	MIC = 64 μg/mL	
				MRSA		
			Cytotoxic[β]	KB	IC_{50} > 74.5 μM	

Table S1. Secondary metabolites of the hyptolactone class (originating from plants or fungi), and their biological effects. (end)

Hypto-lactones	Sources	Families	Biological effects	Biological models	Doses/Concentrations/ Potencies	Studies
**85**	*Mesosphaerum pectinatum* (L.) Kuntze	Lamiaceae	Cytotoxic[β]	MCF-7	$IC_{50} > 10\ \mu M$	Martínez-Fructuoso *et al.*, 2019[28]
				HeLa		
				HCT-15		
**86**	*Mesosphaerum pectinatum* (L.) Kuntze	Lamiaceae	Cytotoxic[β]	MCF-7	$IC_{50} > 10\ \mu M$	Martínez-Fructuoso *et al.*, 2019[28]
				HeLa		
				HCT-15		

[#] The values originally reported as ED_{50} were standardized as IC_{50}. Values originally expressed in µg/mL (except for common concentration or MIC/MFC), µmol/mL, or mM were converted to µM.
[*] Originating from fungi.
[α] *In vivo* biological assay. [β] *In vitro* biological assay. [γ] *In vitro* biological assay, supported by *in silico* study. [δ] *Ex vivo* biological assay.
[a] Cancer cell lines:
Humans = brain (LN229); breast (BC1, MCF-7, MDA-MB-231, NCI-ADR, T47D, and ZR75-1: hormone dependent); cervical (CaSki, HeLa and SQC-1 UISO); colorectal (Col2, HCT-15, HT-29 and LOVO); epidermoid (A431); fibrosarcoma (HT-1080); hepatic (HepG2, Huh7 and MHCC97H); intestine (MGC); kidney (786-0); larynx (Hep-2); lung (A549, Lu1 and NCI 460); melanoma (Mel2 and UACC62); nasopharynx (KB, KB-V: KB vinblastine resistant, KB-V (-VLB) and KB-V (+VLB)); neuroblastoma (SK-N-SH); ovary (OVCAR and OVCAR03); pancreatic (PANC 1); prostate (LNCaP and PC-3).
Murine = lymphocytic leukemia (P-388).
[b] U-87-CCR5 cell, the evaluation was through its conjugated ligands MIP-1α, MIP-1β and RANTES. [c] *Multidrug-resistant Staphylococcus aureus* (MDRSA); *Methicillin-resistant Staphylococcus aureus* (MRSA). [d] Quorum Sensing (QS) inhibitory activity.
[I] Growth inhibition, stress induction and interference with cell wall biosynthesis. [II] Growth inhibition and interference with cell wall biosynthesis. [III] Inhibition in mycelia, mycelia-yeasts transitions and yeasts in the presence of acetate and glucose. [IV] Thin-layer chromatography silica gel plate. [V] Disk-diffusion plate test, antibacterial activity was evaluated by measuring the diameter of the inhibition zone (IZ) around the disks.[VI] Different strains of the species.

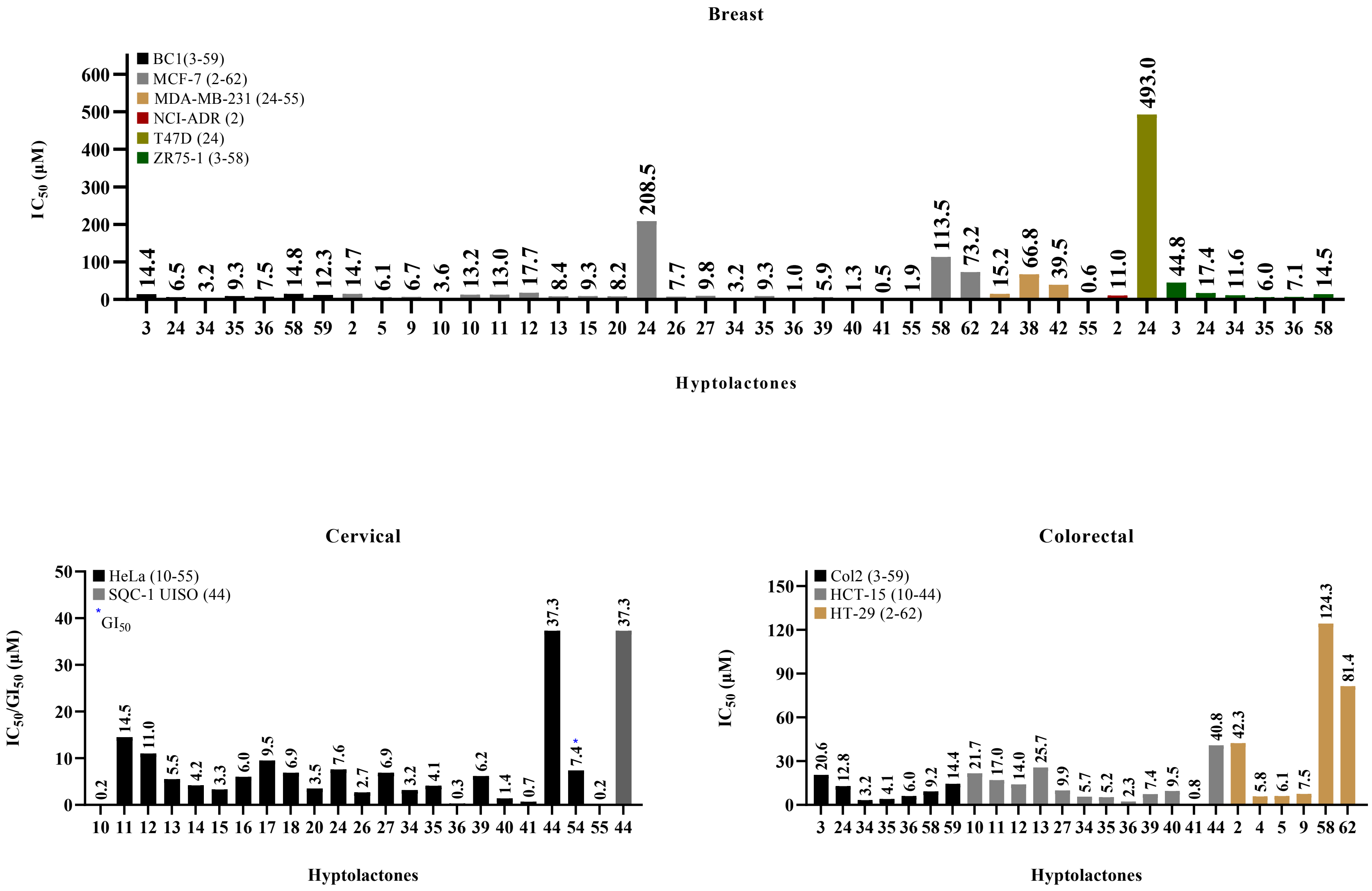

Figure S3. Cytotoxic effects of hyptolactones from the α,β-unsaturated δ-lactone.

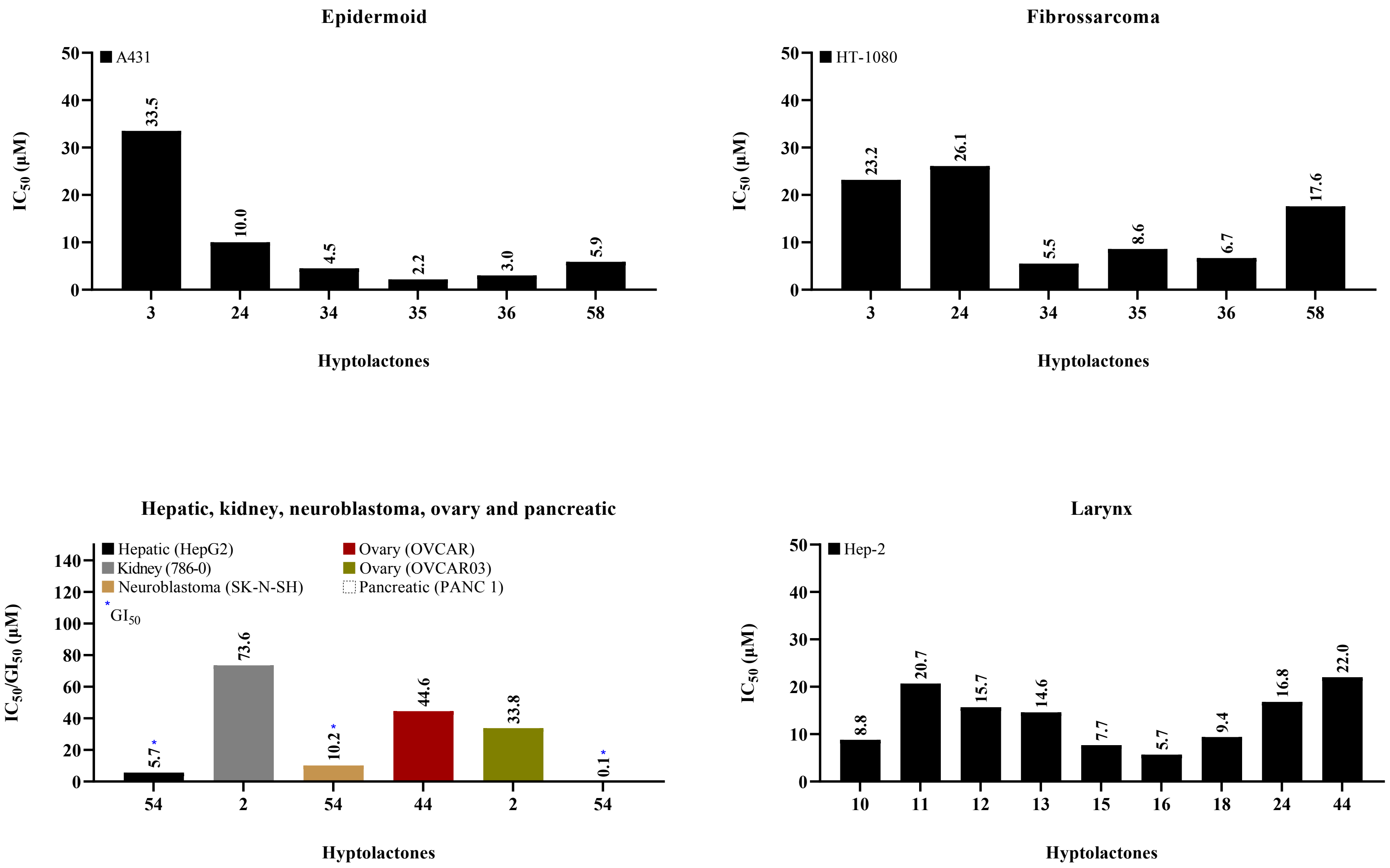

Figure S3. Cytotoxic effects of hyptolactones from the α,β-unsaturated δ-lactone. (cont.)

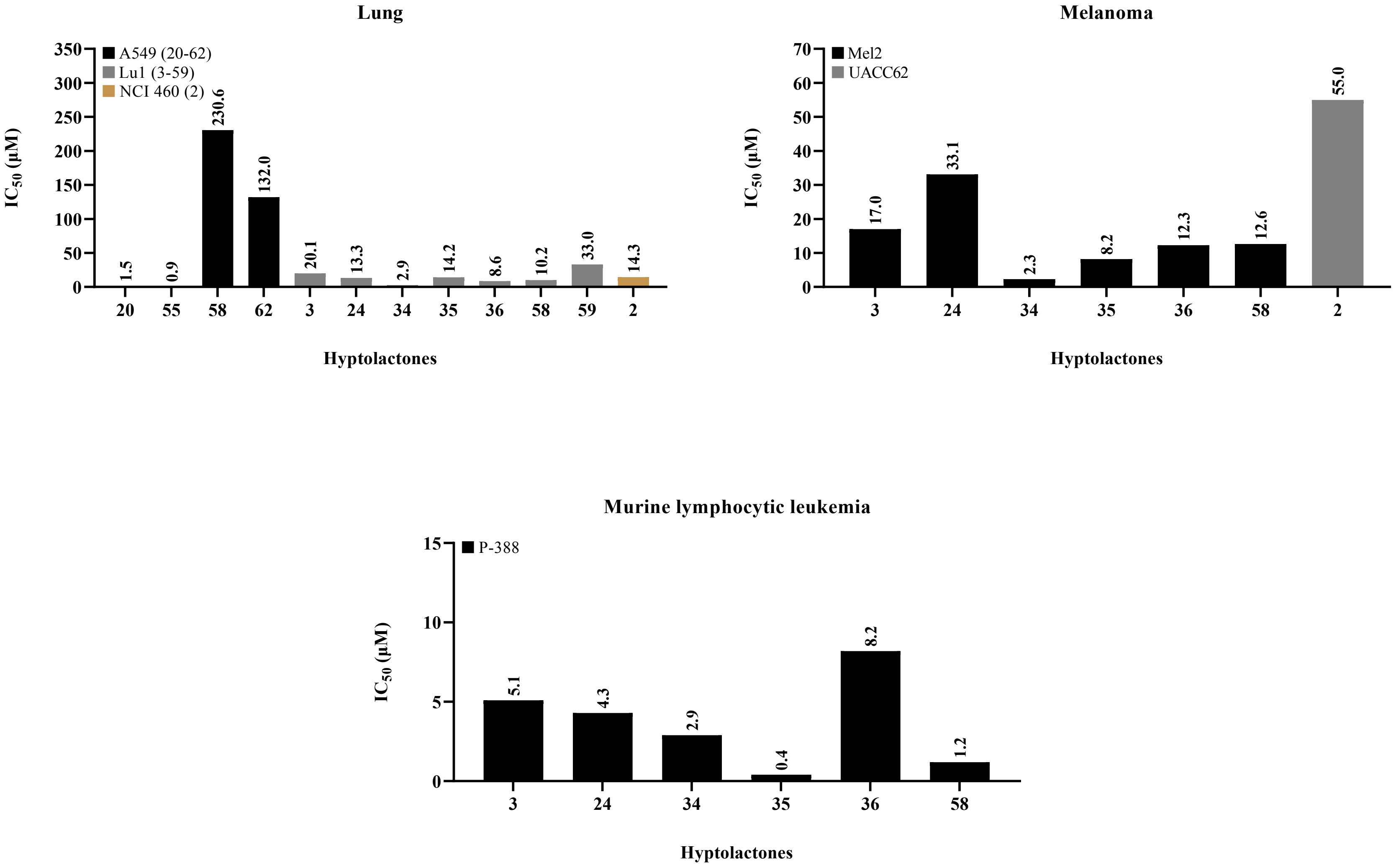

Figure S3. Cytotoxic effects of hyptolactones from the α,β-unsaturated δ-lactone. (cont.)

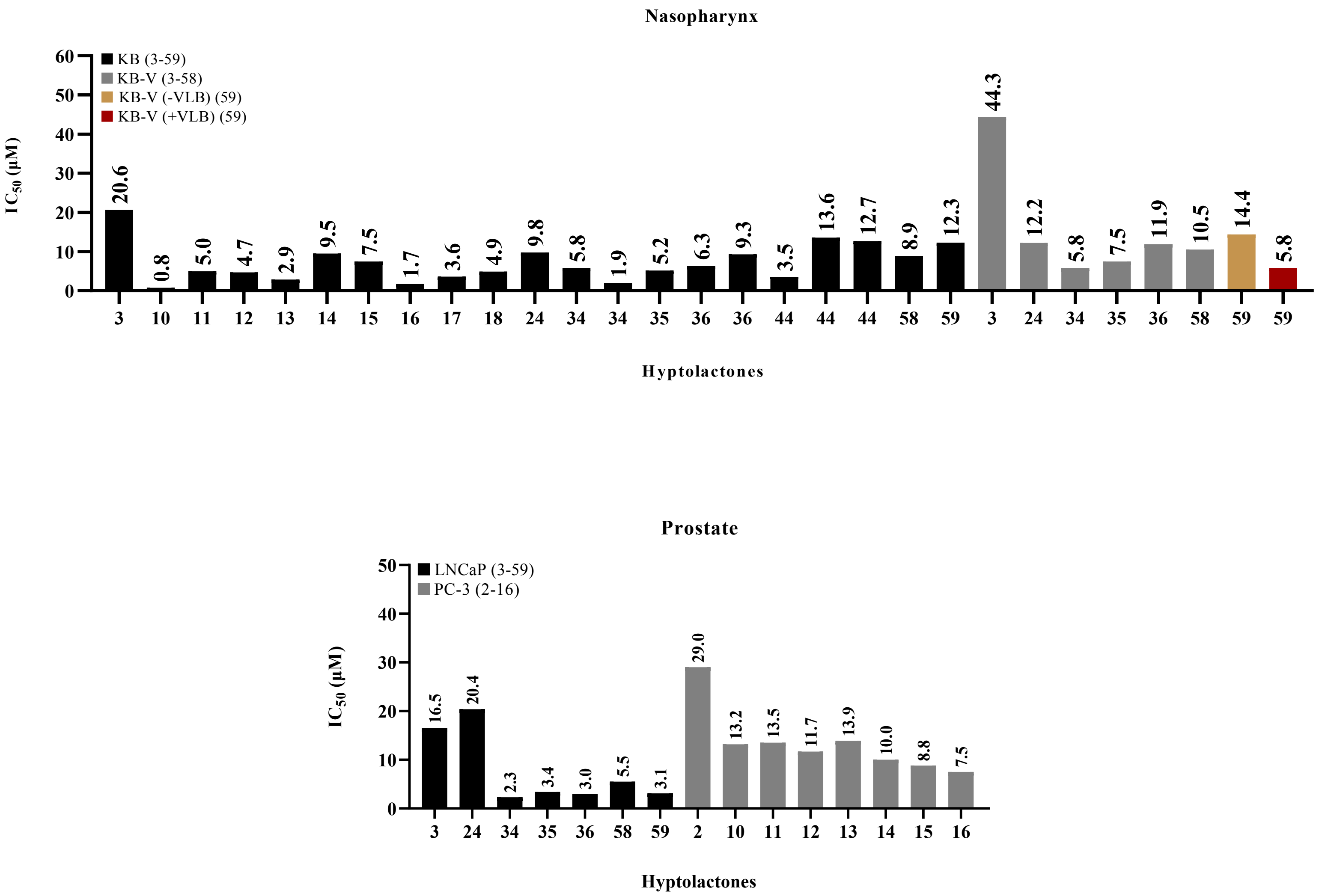

Figure S3. Cytotoxic effects of hyptolactones from the α,β-unsaturated δ-lactone. (end)

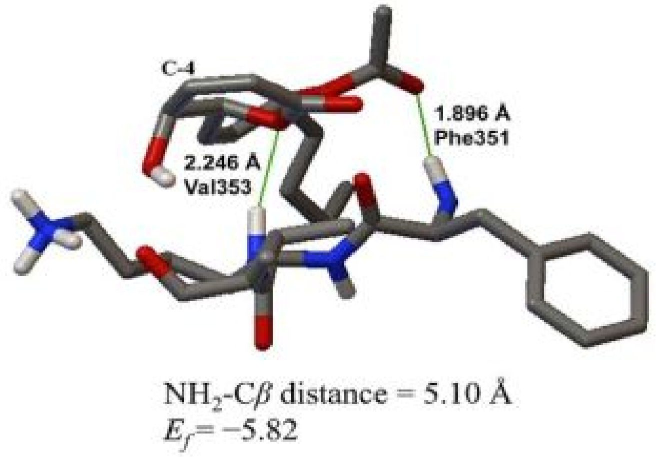

Figure S4. Complex between pectinolide C (**36**) and α-tubulin, showing the distance between the residual amino group of the lysine amino acid (Lys352) and the β-carbon of the α,β-unsaturated lactone (H_2N-Cβ). The binding energy (E_f) values and the H_2N-Cβ bond distance are indicated. Adapted from Martínez-Fructuoso *et al.*, 2019.[28] Copyright 2019 American Chemical Society.

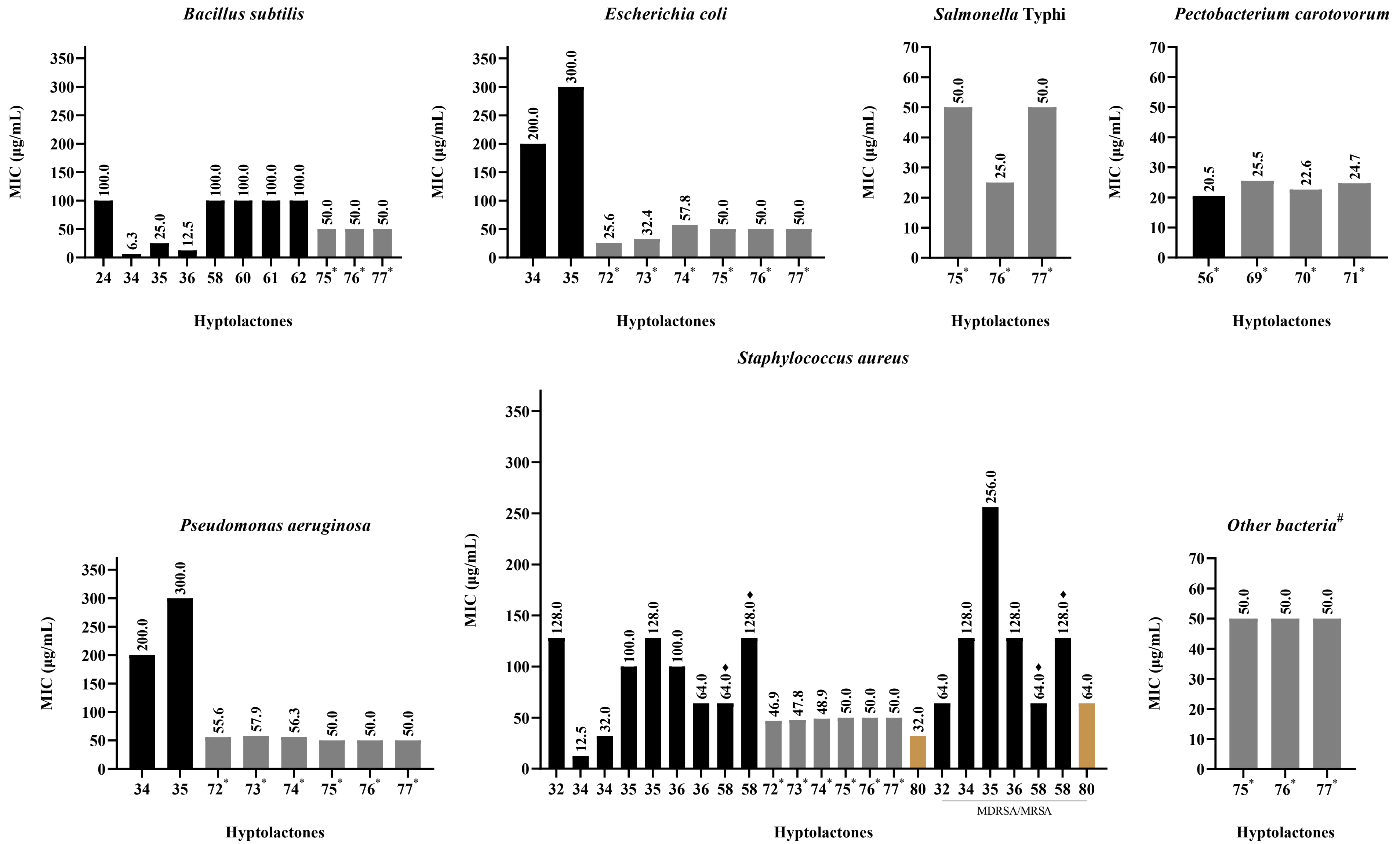

* Isolated from fungi; # *Klebsiella aerogenes*, *Micrococcus luteus*, *Priestia megaterium*, *Proteus vulgaris* and *Salmonella enterica*; ♦ Different strains.

■ α,β-unsaturated δ-lactone ■ α,β,γ,δ-unsaturated δ-lactone ■ α,β-unsaturated γ-lactone

Figure S5. Antibacterial effects of hyptolactones.

Table S2. Assessment of the risk of bias in the study by Costa *et al*., 2014,[21] based on SYRCLE tool guidelines.

**Hyptolactone evaluated**		**Biological effect**	**Biological model**	**Study**
Hyptenolide (**23**)		Antispasmodic	Ileum* and trachea** of *Cavia porcellus*	*Ex vivo*
**Itens**	**Judgment**	**Comments**		
1	Unclear	-		
2	Yes	-		
3	Unclear	-		
4	Unclear	-		
5	Unclear	-		
6	Unclear	-		
7	Unclear	-		
8	Yes	There were no losses.		
9	Yes	All biological assay results reported in the methodology are presented.		
10	Yes	-		

Spasms induced by carbachol and histamine*; and by carbachol**.

Table S3. Assessment of the risk of bias in the study by Waechter *et al*., 1997,[3] based on SYRCLE tool guidelines.

Hyptolactone evaluated		Biological effect	Biological model	Study
Argentilactone (**2**)		Leishmanicidal	Paws and spleens of BALB/c infected with *Leishmania amazonensis*	*In vivo*
**Itens**	**Judgment**	**Comments**		
1	Unclear	-		
2	Yes	Only the species and sex of the animals were reported, however, the time to start treatment after exposure (i.e., the fourth week) was also reported.		
3	Unclear	It was reported that animal allocation was random, however, no information was provided on how randomness was determined		
4	Unclear	-		
5	Unclear	-		
6	Unclear	-		
7	Unclear	-		
8	Yes	There were no losses.		
9	Yes	All biological assay results reported in the methodology are presented.		
10	No	The numerical values presented in the summary differ from those in the tables. Furthermore, the percentage reduction in lesion size reported in the text, is inconsistent with the tabulated data. In the same paragraph, it is stated that the experiment concluded in the sixth week; however, another section of the study reports the endpoint as the seventh week		

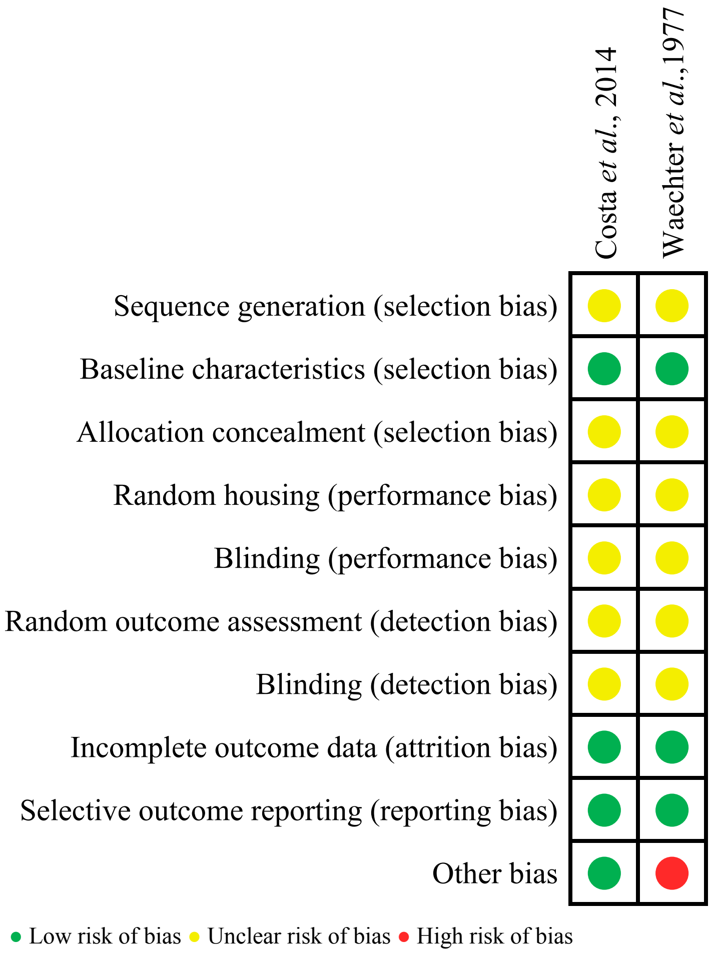

Figure S6. Result of the risk of bias assessment, based on the SYRCLE tool guidelines, applied to the studies by Costa *et al.*, 2014[21] and Waechter *et al.*, 1997.[3]

Table S4. Search strategies used to retrieve records in databases.

Databases	Search strategies
PMC	"δ-lactone" OR "delta-lactone" OR "δ-lactones" OR "delta-lactones" OR "α-pyrone" OR "α-pyrones" AND "5,6-dihydro-α-pyrone" OR "5,6-dihydro-α-pyrones" OR "5,6-dihydro-2H-pyran-2-one" OR "5,6-dihydro-2H-pyran-2-ones" OR "6-heptyl-5,6-dihydro-2H-pyran-2-one" OR "6-heptyl-5,6-dihydro-2H-pyran-2-ones"
ScienceDirect	"δ-lactone" AND "5,6-dihydro-α-pyrone" OR "5,6-dihydro-α-pyrones" OR "5,6-dihydro-2H-pyran-2-one" OR "5,6-dihydro-2H-pyran-2-ones" OR "6-heptyl-5,6-dihydro-2H-pyran-2-one" OR "6-heptyl-5,6-dihydro-2H-pyran-2-ones"
Scopus	"δ-lactone" OR "delta-lactone" OR "δ-lactones" OR "delta-lactones" OR "α-pyrone" OR "α-pyrones" AND "5,6-dihydro-α-pyrone" OR "5,6-dihydro-α-pyrones" OR "5,6-dihydro-2H-pyran-2-one" OR "5,6-dihydro-2H-pyran-2-ones" OR "6-heptyl-5,6-dihydro-2H-pyran-2-one" OR "6-heptyl-5,6-dihydro-2H-pyran-2-ones"
Web of Science	"δ-lactone" OR "δ-lactones" OR "α-pyrone" OR "α-pyrones" AND "5,6-dihydro-α-pyrone" OR "5,6-dihydro-α-pyrones" OR "5,6-dihydro-2H-pyran-2-one" OR "5,6-dihydro-2H-pyran-2-ones" OR "6-heptyl-5,6-dihydro-2H-pyran-2-one" OR "6-heptyl-5,6-dihydro-2H-pyran-2-ones"

Table S5. SYRCLE's tool for assessing risk of bias.

Items	Domain (Type of bias)	Signaling questions
1	Sequence generation (Selection bias)	Was the allocation sequence adequately generated and applied?
2	Baseline characteristics (Selection bias)	Were the groups similar at baseline or were they adjusted for confounders in the analysis?
3	Allocation concealment (Selection bias)	Was the allocation adequately concealed?
4	Random housing (Performance bias)	Were the animals randomly housed during the experiment?
5	Blinding (Performance bias)	Were the caregivers and/or investigators blinded from knowledge which intervention each animal received during the experiment?
6	Random outcome assessment (Detection bias)	Were animals selected at random for outcome assessment?
7	Blinding (detection bias)	Was the outcome assessor blinded?
8	Incomplete outcome data (Attrition bias)	Were incomplete outcome data adequately addressed?
9	Selective outcome reporting (Reporting bias)	Are reports of the study free of selective outcome reporting?
10	Other bias	Is the study free from textual divergences, numbers, symbols or measurement units between the experimental procedure and results?*

*Prepared by the authors of the systematic review.

REFERENCES

(1) Yang, J.; Wang, Y.; Wang, T.; Jiang, J.; Botting, C. H.; Liu, H.; Chen, Q.; Yang, J.; Naismith, J. H.; Zhu, X.; Chen, L. Pironetin reacts covalently with cysteine-316 of α-tubulin to destabilize microtubule. *Nat. Commun.* **2016**, *7* (1), 1–9. https://doi.org/10.1038/ncomms12103.

(2) Wang, Y.; Naismith, J.; Zhu, X. 5FNV: A new complex structure of tubulin with an alpha-beta unsaturated lactone. RCSB Protein Data Bank. https://www.rcsb.org/structure/5FNV (accessed 2025-10-01).

(3) Waechter, A.; Ferreira, M.; Fournet, A.; de Arias, A.; Nakayama, H.; Torres, S.; Hocquemiller, R.; Cavé, A. Experimental treatment of cutaneous leishmaniasis with argentilactone isolated from *Annona Haematantha*. *Planta Med.* **1997**, *63* (05), 433–435. https://doi.org/10.1055/s-2006-957728.

(4) Carmona, D.; Sáez, J.; Granados, H.; Pérez, E.; Blair, S.; Angulo, A.; Figadere, B. Antiprotozoal 6-substituted-5,6-dihydro-a-pyrones from *Raimondia* cf. *monoica*. *Nat. Prod. Res.* **2003**, *17* (4), 275–280. https://doi.org/10.1080/1057563031000065062.

(5) de Oliveira, C. M. A.; Silva, M. do R. R.; Kato, L.; Silva, C. C. da; Ferreira, H. D.; Souza, L. K. H. Chemical composition and antifungal activity of the essential oil of *Hyptis ovalifolia* Benth. (Lamiaceae). *J. Braz. Chem. Soc.* **2004**, *15* (5), 756–759. https://doi.org/10.1590/S0103-50532004000500023.

(6) Prado, R. S.; Alves, R. J.; de Oliveira, C. M. A.; Kato, L.; da Silva, R. A.; Quintino, G. O.; Cunha, S. do D.; Soares, C. M. de A.; Pereira, M. Inhibition of *Paracoccidioides lutzii* Pb01 isocitrate lyase by the natural compound argentilactone and its semi-synthetic derivatives. *PLoS One* **2014**, *9* (4), 1–13. https://doi.org/10.1371/journal.pone.0094832.

(7) Prado, R. S.; Bailão, A. M.; Silva, L. C.; de Oliveira, C. M. A.; Marques, M. F.; Silva, L. P.; Silveira-Lacerda, E. P.; Lima, A. P.; Soares, C. M.; Pereira, M. Proteomic profile response of *Paracoccidioides lutzii* to the antifungal argentilactone. *Front. Microbiol.* **2015**, *6*, 1–14. https://doi.org/10.3389/fmicb.2015.00616.

(8) Araújo, F. S.; Coelho, L. M.; Silva, L. do C.; Neto, B. R. da S.; Parente-Rocha, J. A.; Bailão, A. M.; de Oliveira, C. M. A.; Fernandes, G. da R.; Hernández, O.; Ochoa, J. G. M.; Soares, C. M. de A.;

Pereira, M. Effects of argentilactone on the transcriptional profile, cell wall and oxidative stress of *Paracoccidioides* spp. *PLoS Negl. Trop. Dis.* **2016**, *10* (1), 1–18. https://doi.org/10.1371/journal.pntd.0004309.

(9) Silva, L. do C.; Tauhata, S. B. F.; Baeza, L. C.; de Oliveira, C. M. A.; Kato, L.; Borges, C. L.; Soares, C. M. de A.; Pereira, M. Argentilactone molecular targets in *Paracoccidioides brasiliensis* identified by chemoproteomics. *Antimicrob. Agents Chemother.* **2018**, *62* (11), 1–13. https://doi.org/10.1128/AAC.00737-18.

(10) Saeed, M.; Ilg, T.; Schick, M.; Abbas, M.; Voelter, W. Total synthesis and anti-leishmanial activity of *R*-(−)-argentilactone. *Tetrahedron Lett.* **2001**, *42* (42), 7401–7403. https://doi.org/10.1016/S0040-4039(01)01559-3.

(11) de Fátima, Â.; Marquissolo, C.; de Albuquerque, S.; Carraro-Abrahão, A. A.; Pilli, R. A. Trypanocidal activity of 5,6-dihydropyran-2-ones against free trypomastigotes forms of *Trypanosoma cruzi*. *Eur. J. Med. Chem.* **2006**, *41* (10), 1210–1213. https://doi.org/10.1016/j.ejmech.2006.05.010.

(12) de Fatima, Â.; Kohn, L. K.; Antônio, M. A.; de Carvalho, J. E.; Pilli, R. A. Enantioselective syntheses of (*R*)- and (*S*)-argentilactone and their cytotoxic activities against cancer cell lines. *Bioorg. Med. Chem.* **2004**, *12* (20), 5437–5442. https://doi.org/10.1016/j.bmc.2004.07.044.

(13) Yamauchi, S.; Isozaki, Y.; Nishimura, H.; Tsuda, T.; Nishiwaki, H.; Shuto, Y. Total syntheses of (−)- and (+)-boronolide and their plant growth-inhibitory activity. *Biosci. Biotechnol. Biochem.* **2012**, *76* (9), 1708–1714. https://doi.org/10.1271/bbb.120317.

(14) Pereda-Miranda, R.; Hernández, L.; Villavicencio, M. J.; Novelo, M.; Ibarra, P.; Chai, H.; Pezzuto, J. M. Structure and stereochemistry of pectinolides A-C, novel antimicrobial and cytotoxic 5,6-dihydro-α-pyrones from *Hyptis pectinata*. *J. Nat. Prod.* **1993**, *56* (4), 583–593. https://doi.org/10.1021/np50094a019.

(15) Deng, Y.; Balunas, M. J.; Kim, J.-A.; Lantvit, D. D.; Chin, Y.-W.; Chai, H.; Sugiarso, S.; Kardono, L. B. S.; Fong, H. H. S.; Pezzuto, J. M.; Swanson, S. M.; de Blanco, E. J. C.; Kinghorn, A. D. Bioactive 5,6-dihydro-α-pyrone derivatives from *Hyptis brevipes*. *J. Nat. Prod.* **2009**, *72* (6), 1165–

1169. https://doi.org/10.1021/np9001724.

(16) Hegde, V. R.; Pu, H.; Patel, M.; Das, P. R.; Strizki, J.; Gullo, V. P.; Chou, C.-C.; Buevich, A. V.; Chan, T.-M. Three new compounds from the plant Lippia alva as inhibitors of chemokine receptor 5 (CCR5). *Bioorg. Med. Chem. Lett.* **2004**, *14* (21), 5339–5342. https://doi.org/10.1016/j.bmcl.2004.08.021.

(17) Suárez-Ortiz, G. A.; Cerda-García-Rojas, C. M.; Hernández-Rojas, A.; Pereda-Miranda, R. Absolute configuration and conformational analysis of brevipolides, bioactive 5,6-dihydro-α-pyrones from *Hyptis brevipes*. *J. Nat. Prod.* **2013**, *76* (1), 72–78. https://doi.org/10.1021/np300740h.

(18) Suárez-Ortiz, G. A.; Cerda-García-Rojas, C. M.; Fragoso-Serrano, M.; Pereda-Miranda, R. Complementarity of DFT calculations, NMR anisotropy, and ECD for the configurational analysis of brevipolides K-O from *Hyptis brevipes*. *J. Nat. Prod.* **2017**, *80* (1), 181–189. https://doi.org/10.1021/acs.jnatprod.6b00953.

(19) Sabitha, G.; Reddy, D. V.; Reddy, S. S. S.; Yadav, J. S.; Kumar, C. G.; Sujitha, P. Total synthesis of desacetylumuravumbolide, umuravumbolide and their biological evaluation. *RSC Adv.* **2012**, *2* (18), 7241–7247. https://doi.org/10.1039/c2ra20830j.

(20) Koshino, H.; Yoshihara, T.; Okuno, M.; Sakamura, S.; Tajimi, A.; Shimanuki, T. Gamahonolides A, B, and gamahorin, novel antifungal compounds from stromata of *Epichloe typhina* on *Phleum pratense*. *Biosci. Biotechnol. Biochem.* **1992**, *56* (7), 1096–1099. https://doi.org/10.1271/bbb.56.1096.

(21) Costa, V. C. de O.; Tavares, J. F.; Silva, A. B.; Duarte, M. C.; Agra, M. de F.; Barbosa-Filho, J. M.; de Souza, I. L. L.; da Silva, B. A.; Silva, M. S. Hyptenolide, a new α-pyrone with spasmolytic activity from *Hyptis macrostachys*. *Phytochem. Lett.* **2014**, *8*, 32–37. https://doi.org/10.1016/j.phytol.2014.01.009.

(22) Rojas, A.; Hernandez, L.; Pereda-Miranda, R.; Mata, R. Screening for antimicrobial activity of crude drug extracts and pure natural products from mexican medicinal plants. *J. Ethnopharmacol.* **1992**, *35* (3), 275–283. https://doi.org/10.1016/0378-8741(92)90025-M.

(23) Bambang, C.; Meiny, S.; Nur Dina, A.; Wahyudi, W.; Damar Nurwahyu, B. Synthesis and

antibacterial activity of epoxide from hyptolide (*Hyptis pectinata* (L.) Poit) against Gram-positive and Gram-negative bacteria. *J. Appl. Pharm. Sci.* **2020**, *10* (12), 13–22. https://doi.org/10.7324/JAPS.2020.101202.

(24) Suzery, M.; Cahyono, B.; Amalina, N. D. Antiproliferative and apoptosis effect of hyptolide from *Hyptis pectinata* (L.) Poit on human breast cancer cells. *J. Appl. Pharm. Sci.* **2020**, *10* (2), 1–6. https://doi.org/10.7324/JAPS.2020.102001.

(25) Santana, F. R.; Luna-Dulcey, L.; Antunes, V. U.; Tormena, C. F.; Cominetti, M. R.; Duarte, M. C.; da Silva, J. A. Evaluation of the cytotoxicity on breast cancer cell of extracts and compounds isolated from *Hyptis pectinata* (L.) Poit. *Nat. Prod. Res.* **2019**, *34* (1), 102–109. https://doi.org/10.1080/14786419.2019.1628747.

(26) Mendoza-Espinoza, J. A.; López-Vallejo, F.; Fragoso-Serrano, M.; Pereda-Miranda, R.; Cerda-García-Rojas, C. M. Structural reassignment, absolute configuration, and conformation of hypurticin, a highly flexible polyacyloxy-6-heptenyl-5,6-dihydro-2*H*-pyran-2-one. *J. Nat. Prod.* **2009**, *72* (4), 700–708. https://doi.org/10.1021/np800447k.

(27) Ludere, M. T.; van Ree, T.; Vleggaar, R. Isolation and relative stereochemistry of lippialactone, a new antimalarial compound from *Lippia javanica*. *Fitoterapia* **2013**, *86*, 188–192. https://doi.org/10.1016/j.fitote.2013.03.009.

(28) Martínez-Fructuoso, L.; Pereda-Miranda, R.; Rosas-Ramírez, D.; Fragoso-Serrano, M.; Cerda-García-Rojas, C. M.; da Silva, A. S.; Leitão, G. G.; Leitão, S. G. Structure elucidation, conformation, and configuration of cytotoxic 6-heptyl-5,6-dihydro-2*H*-pyran-2-ones from *Hyptis* species and their molecular docking to α-tubulin. *J. Nat. Prod.* **2019**, *82* (3), 520–531. https://doi.org/10.1021/acs.jnatprod.8b00908.

(29) Rahman, M. M.; Gibbons, S. Antibacterial constituents of *Neohyptis paniculata*. *Fitoterapia* **2015**, *105*, 269–272. https://doi.org/10.1016/j.fitote.2015.07.012.

(30) Fragoso-Serrano, M.; Gibbons, S.; Pereda-Miranda, R. Anti-staphylococcal and cytotoxic compounds from *Hyptis pectinata*. *Planta Med.* **2005**, *71* (3), 278–280. https://doi.org/10.1055/s-2005-837831.

(31) Pereda-Miranda, R.; Fragoso-Serrano, M.; Cerda-García-Rojas, C. M.; Davies-Coleman, M. T.; Rivett, D. E. A. application of molecular mechanics in the total stereochemical elucidation of spicigerolide, a cytotoxic 6-tetraacetyloxyheptenyl-5,6-dihydro-α-pyrone from *Hyptis spicigera*. *Tetrahedron* **2001**, *57* (1), 47–53. https://doi.org/10.1016/S0040-4020(00)00987-X.

(32) Falomir, E.; Murga, J.; Ruiz, P.; Carda, M.; Marco, J. A.; Pereda-Miranda, R.; Fragoso-Serrano, M.; Cerda-García-Rojas, C. M. Stereoselective synthesis and determination of the cytotoxic properties of spicigerolide and three of its stereoisomers. *J. Org. Chem.* **2003**, *68* (14), 5672–5676. https://doi.org/10.1021/jo034470y.

(33) Sabitha, G.; Rao, A. S.; Sandeep, A.; Latha, B. M.; Reddy, D. V. Total synthesis of (−)-synrotolide and the evaluation of its antiproliferative activity. *Tetrahedron: Asymmetry* **2014**, *25* (10–11), 856–859. https://doi.org/10.1016/j.tetasy.2014.04.010.

(34) Guo, Z.-Y.; Lu, L.-W.; Bao, S.-S.; Liu, C.-X.; Deng, Z.-S.; Cao, F.; Liu, S.-P.; Zou, K.; Proksch, P. Xylariaopyrones A-D, four new antimicrobial α-pyrone derivatives from endophytic fungus *Xylariales* sp. *Phytochem. Lett.* **2018**, *28*, 98–103. https://doi.org/10.1016/j.phytol.2018.09.014.

(35) Pereda-Miranda, R.; García, M.; Delgado, G. Structure and stereochemistry of four α-pyrones from *Hyptis oblongifolia*. *Phytochemistry* **1990**, *29* (9), 2971–2974. https://doi.org/10.1016/0031-9422(90)87117-D.

(36) Lu, G.-H.; Wang, F.-P.; Pezzuto, J. M.; Tam, T. C. M.; Williams, I. D.; Che, C.-T. 10-*epi*-olguine from *Rabdosia ternifolia*. *J. Nat. Prod.* **1997**, *60* (4), 425–427. https://doi.org/10.1021/np960689e.

(37) Gao, H.; Li, G.; Peng, X.-P.; Lou, H.-X. Fupyrones A and B, two new α-pyrones from an endophytic fungus, *Fusarium* sp. F20. *Nat. Prod. Res.* **2020**, *34* (3), 335–340. https://doi.org/10.1080/14786419.2018.1531405.

(38) Yang, W.-W.; Lu, L.-W.; Zhang, X.-Q.; Bao, S.-S.; Cao, F.; Guo, Z.-Y.; Deng, Z.-S.; Proksch, P. Xylariaopyrones E–I, five new α-pyrone derivatives from the endophytic fungus *Xylariales* sp. (HM-1). *Nat. Prod. Res.* **2020**, 1–9. https://doi.org/10.1080/14786419.2020.1826480.

(39) Zhao, T.; Xu, L.-L.; Zhang, Y.; Lin, Z.-H.; Xia, T.; Yang, D.-F.; Chen, Y.-M.; Yang, X.-L. Three new α-pyrone derivatives from the plant endophytic fungus *Penicillium ochrochloronthe* and their

antibacterial, antifungal, and cytotoxic activities. *J. Asian Nat. Prod. Res.* **2018**, *21* (9), 851–858. https://doi.org/10.1080/10286020.2018.1495197.
